# Urothelial Tight Junction Barrier Dysfunction Sensitizes Bladder Afferents

**DOI:** 10.1523/ENEURO.0381-16.2017

**Published:** 2017-05-24

**Authors:** Nicolas Montalbetti, Anna C. Rued, Stefanie N. Taiclet, Lori A. Birder, F. Aura Kullmann, Marcelo D. Carattino

**Affiliations:** 1 Renal-Electrolyte Division, Department of Medicine, University of Pittsburgh, Pittsburgh, PA 15261; 2 Department of Pharmacology and Chemical Biology, University of Pittsburgh, Pittsburgh, PA 15261; 3 Department of Cell Biology, University of Pittsburgh, Pittsburgh, PA 15261

**Keywords:** afferent innervations, DRG neurons, interstitial cystitis/bladder pain syndrome, pain, tetrodotoxin, urinary bladder

## Abstract

Interstitial cystitis/bladder pain syndrome (IC/BPS) is a chronic voiding disorder that presents with pain in the urinary bladder and surrounding pelvic region. A growing body of evidence suggests that an increase in the permeability of the urothelium, the epithelial barrier that lines the interior of the bladder, contributes to the symptoms of IC/BPS. To examine the consequence of increased urothelial permeability on pelvic pain and afferent excitability, we overexpressed in the urothelium claudin 2 (Cldn2), a tight junction (TJ)-associated protein whose message is significantly upregulated in biopsies of IC/BPS patients. Consistent with the presence of bladder-derived pain, rats overexpressing Cldn2 showed hypersensitivity to von Frey filaments applied to the pelvic region. Overexpression of Cldn2 increased the expression of c-Fos and promoted the activation of ERK1/2 in spinal cord segments receiving bladder input, which we conceive is the result of noxious stimulation of afferent pathways. To determine whether the mechanical allodynia observed in rats with reduced urothelial barrier function results from altered afferent activity, we examined the firing of acutely isolated bladder sensory neurons. In patch-clamp recordings, about 30% of the bladder sensory neurons from rats transduced with Cldn2, but not controls transduced with GFP, displayed spontaneous activity. Furthermore, bladder sensory neurons with tetrodotoxin-sensitive (TTX-S) action potentials from rats transduced with Cldn2 showed hyperexcitability in response to suprathreshold electrical stimulation. These findings suggest that as a result of a leaky urothelium, the diffusion of urinary solutes through the urothelial barrier sensitizes bladders afferents, promoting voiding at low filling volumes and pain.

## Significance Statement

Interstitial cystitis/bladder pain syndrome (IC/BPS) is a chronic voiding disorder with symptoms that include urinary urgency, urinary frequency, and pain in the bladder and surrounding pelvic region, in the absence of proven urinary infection or other noticeable pathology. Although the exact cause of this disorder is unknown, numerous lines of evidence suggest that changes in the permeability of the epithelial cell layer that cover the internal surface of the urinary bladder contribute to the perpetuation of the symptoms. The present study examines the mechanisms that mediate lower urinary tract symptoms and pain in an animal model with reduced urothelial barrier function.

## Introduction

Interstitial cystitis/bladder pain syndrome (IC/BPS) is a chronic voiding disorder that presents with pain in the bladder and surrounding pelvic region (Nickel, 2002; Moutzouris and Falagas, 2009; Hanno et al., 2011; Neuhaus and Schwalenberg, 2012; Birder, 2014). While the cause of IC/BPS is unknown, several lines of evidence indicate that increased urothelial permeability to urine constituents plays an important role in the pathophysiology of this disease (Parsons et al., 1991; Chai and Keay, 2004; Graham and Chai, 2006; Parsons, 2007, 2011). The internal surface of the urinary bladder is lined by the urothelium, a stratified epithelium that restricts the passage of ions and metabolic products from the urine into the bladder interstitium (Khandelwal et al., 2009). Because bladder afferent terminals reside within the urothelium and in a subepithelial plexus very close to the basal surface of the epithelium (Gabella and Davis, 1998), changes in the permeability of the urothelial barrier to urinary constituents can alter sensory input and elicit painful sensations. Consistent with the presence of increased bladder afferent input, IC/BPS patients present pain that augments with bladder filling and is relieved by bladder emptying (Nickel, 2002; Moutzouris and Falagas, 2009; Hanno et al., 2011; Neuhaus and Schwalenberg, 2012).

The urinary bladder is innervated by the pelvic and hypogastric nerves, which convey information about the filling status and noxious stimuli to the CNS (de Groat and Yoshimura, 2009). These nerves consist of small myelinated Aδ fibers that respond to passive distension of the bladder wall and active contraction, carrying information about bladder filling, and unmyelinated C fibers with high mechanical threshold that respond to noxious chemical stimulation of the bladder mucosa (de Groat and Yoshimura, 2009). A population of fibers with a conduction speed of C afferents that responds to bladder distension in the physiologic range has also been described (Floyd et al., 1976; Bahns et al., 1986; Sengupta and Gebhart, 1994; Wen and Morrison, 1995; Dmitrieva and McMahon, 1996). While strong evidence indicates that afferent sensitization contributes to voiding dysfunction and pain in IC/BPS (Daly et al., 2011; Gonzalez et al., 2014; Yoshimura et al., 2014; Ogawa et al., 2015), the type of afferents affected and the molecular mechanism of sensitization remain largely unknown.

Although the urothelium is multilayered, it is the outermost umbrella cell layer that forms a multifactorial barrier that includes a mucin layer with anti-adherence properties, an apical membrane with inherently low permeability to urea and water, and relatively impermeable tight junctions (TJs; Khandelwal et al., 2009). There is a strong and growing body of evidence showing changes in the expression of TJ-associated proteins in biopsies from patients with IC/BPS (Slobodov et al., 2004; Hauser et al., 2008; Sanchez Freire et al., 2010; Lee and Lee, 2014; Hauser et al., 2015). TJs consist of a network of cytosolic and membrane proteins that associate to seal the paracellular space between adjacent epithelial cells just beneath their apical surface. Claudins (Cldns), a family of tetra-membrane spanning proteins, form the structural and functional core of the TJs and thus define the electrical properties of epithelia. Recently, an extensive analysis of gene expression reported a ninety-fold upregulation of Cldn2 mRNA levels in biopsies of patients with IC/BPS, when compared with controls (Sanchez Freire et al., 2010). In support of the notion that Cldn2 upregulation contributes to IC/BPS symptoms, we recently showed that the overexpression of Cldn2 in the umbrella cell layer increases the permeability of the urothelium to small ions, triggers an inflammatory process in the bladder mucosa and lamina propria, and increases voiding frequency (Montalbetti et al., 2015).

The present work examines the consequences of Cldn2 overexpression in the urothelium on somatic sensitivity in the pelvic area and bladder afferent firing. The results of our studies indicate that Cldn2 upregulation reduces pelvic mechanical threshold by sensitizing primarily bladders afferents of Aδ origin.

## Materials and Methods

### Reagents and antibodies

All chemicals were purchased from Sigma-Aldrich, unless otherwise specified. Mouse monoclonal antibody to Cldn2 was purchased from Invitrogen, mouse monoclonal antibody to β-actin was from Sigma and rabbit polyclonal to c-Fos was from EnCor Biotechnology. Monoclonal mouse to phospho-p44/42 MAPK (pERK1/2) (Thr202/Tyr204) antibody and rabbit p44/42 MAPK (ERK1/2) antibody were purchased from Cell Signaling Technology. Cy3-coupled, FITC-coupled, biotinylated donkey anti-rabbit IgG, and horseradish peroxidase-coupled secondary antibodies were purchased from Jackson ImmunoResearch. Phalloidin Rhodamine- and Phalloidin Alexa Fluor 647-conjugated were purchased from Invitrogen.

### Animals

All experimental procedures were approved by the University of Pittsburgh Institutional Animal Care and Use Committee. Female Sprague Dawley rats (250–300 g; Envigo) were used throughout. Rats were housed under a 12/12 h light/dark cycle with free access to food and water. Animals were randomized to blinded treatment and control groups. The stage of the estrous cycle was not monitored. Animals were euthanized by CO_2_ inhalation, followed by a thoracotomy.

### Preparation of recombinant adenoviruses and *in situ* transduction of umbrella cells

Replication-defective adenoviruses coding for GFP (AdGFP) or Cldn2 (AdCldn2) were constructed by subcloning the coding region of the respective proteins into the plasmid pAdlox as previously described (Montalbetti et al., 2015). Adenoviruses were generated at the University of Pittsburgh Vector Core facility. *In situ* transduction of umbrella cells was accomplished via intravesical instillation of adenoviruses under isoflurane anesthesia (Khandelwal et al., 2008; Montalbetti et al., 2015). Briefly, a day before physiologic studies were conducted a 22-gauge Teflon catheter (Smiths Medical) was inserted in the urethra to drain the urine and wash the urinary bladder. After three consecutive washes with 450 μl of Dulbecco’s PBS buffer (DPBS), the bladder was infused with 450 μl of 0.1% (w/v) n-Dodecyl-β-D-maltoside dissolved in DPBS. The catheter was removed immediately after, and the urethral orifice was clamped with a metal clip to prevent leakage. The metal clip was applied to the skin and musculature surrounding the urethra. After 5 min, the catheter was reintroduced through the urethra and the bladder was emptied. The urinary bladder was infused thereafter with 450 μl of DPBS containing 2 × 10^10^ infectious virus particles of AdGFP or AdCldn2 and the external urethral orifice was clamped again. After a 30 min incubation, the catheter was reintroduced through the urethra and the bladder was emptied and washed one time with DPBS. Then, rats were allowed to recover from anesthesia. No signs of pain due to urethral clamping (i.e., licking or scratching of the pelvic area) were apparent in the transduced animals. Experiments were performed 24 h after adenoviral transduction, unless otherwise indicated.

### Immunofluorescent labeling and image capture

Urinary bladders, urethras and dorsal root ganglia (DRGs) harvested from rats transduced with AdGFP or AdCldn2 were processed as previously described (Montalbetti et al., 2015). Briefly, urinary bladders and urethras were carefully cut open and pinned mucosal side up onto a rubber sheet submerged in Krebs solution containing 110 mM NaCl, 25 mM NaHCO_3_, 5.8 mM KCl, 1.2 mM MgSO_4_, 1.2 mM KH_2_PO_4_, 2 mM CaCl_2_, and 11 mM glucose buffered at pH 7.4 by gassing with a mixture of 95% O_2_/5% CO_2_ (v/v) at 37°C. Bladders, urethras and DRGs were fixed with 4% (v/v) paraformaldehyde in Krebs buffer for 30 min at 37°C. Fixed tissues were incubated in 30% (w/v) sucrose dissolved in PBS at 4°C until the tissue lost its buoyancy and sank to the bottom of the tube. Bladders and urethras were embedded in optimal cutting temperature (O.C.T.) compound. Fixed DRGs were embedded in a 50/50 mixture of O.C.T. and sucrose (30% w/v in PBS), before storage at −80°C. Frozen tissue blocks were sectioned with CM1950 cryostat (Leica). Bladder sections were labeled with primary antibodies or fluorophore-labeled probes as previously described (Montalbetti et al., 2015). Confocal images from bladder and urethra sections were captured using a Leica TCS SP5 CW-STED confocal microscope (in normal confocal mode) equipped with a PL Apo 20× air (N.A. = 0.7) or a PL Apo 63× glycerol objective (N.A. = 1.3) and low-noise hybrid detectors. Images from fixed DRGs were captured with a Leica DM6000B upright microscope (fitted with a 40× HCX PL-APO, 1.25 N.A., objective) equipped with a QImaging Retiga 4000R color digital camera interfaced with an Apple iMac computer running Volocity Acquisition software (version 6.3). The captured confocal images were contrast corrected using Volocity (PerkinElmer) or ImageJ (NIH) and then assembled in Adobe Illustrator.

### Measurements of urinary bladder electrical resistance

Urinary bladders harvested from transduced animals were mounted on custom fabricated Teflon tissue sliders with an exposed tissue area of 0.65 cm^2^ and eight sharp pins set at 3 mm from the opening, as previously described (Montalbetti et al., 2015). Silicone grease (Dow Corning) was carefully applied to the region of tissue impaled by the pins to prevent edge damage. Tissue sliders were inserted into the chambers of an EM-CSYS Ussing system (Physiologic Instruments) equipped with a heating block for temperature control. The mucosal and serosal hemichambers were filled with 3 and 5 ml of Krebs solution, respectively. The hemichambers were continuously bubbled with 95% O_2_/5% CO_2_ (v/v) and the temperature inside was kept at 37°C. The mucosal and serosal hemichambers were connected to Ag/AgCl electrodes via 5 M NaCl agar bridges for voltage sensing and current passing. These electrodes were connected to a VCC MC6 Multichannel Voltage/Current Clamp (Physiologic Instruments). Signals were low-pass filtered at 1 kHz (four-pole Bessel filter) and digitized with a Digidata 1440A interface at 5 kHz (Molecular Devices). Command protocols and data acquisition were controlled by pClamp 10 (Molecular Devices). Liquid junction potentials were compensated using an offset-removal circuit before tissue mounting. Current/voltage (I/V) relationships were generated by applying 1 μA current steps from −5 to + 5 μA with a duration of 400 ms. Tissue electrical resistance (TER) was calculated from the slope of the curve.

### Tissue edema quantification

To measure bladder edema, the percentage of tissue water content of bladders transduced with AdGFP or AdCldn2 was estimated (Carattino et al., 2000). Urinary bladders were harvested through an abdominal incision, carefully dried with a Kimwipe paper to eliminate the urine and weighed to obtain the wet mass. To determine the dry mass, samples were desiccated to constant weight at 55°C. The percentage of water tissue content was calculated as,

$$\frac{WM-DM}{WM}\times 100$$

### Assessment of mechanical allodynia

Thresholds to mechanical stimuli applied to the pelvic area and hind paw were estimated with von Frey filaments (Touch Test Sensory Evaluators, North Coast Medical) using the up-down method described by Chaplan and colleagues (Chaplan et al., 1994). Rats were individually placed in modular cages (Bioseb) on an elevated wire-mesh platform to allow access to the pelvic area and plantar surface of hind paws. The animals were acclimatized for at least 1h before the test. The stimulus was applied on the plantar surface of a hind paw and on the lower abdominal area close to the urinary bladder. von Frey filaments were applied to the tested area for 1–3 s with intervals between stimuli of 15 s. Testing in the pelvic area was initiated with a von Frey filament with a calibrated force of 1 g. Testing in the hind paw was initiated with a von Frey filament with a calibrated force of 10 g. When a negative response was observed, the next-stronger filament was applied. When a positive response was observed, the next-weaker stimulus was applied. Abdominal withdrawal (either contraction of the abdominal musculature or postural retraction of the abdomen), licking or scratching in the pelvic area in response to von Frey filament application were considered a positive response. When stimuli were applied to the plantar surface, a response was considered positive when the animal withdrew the paw sharply or licked the tested limb. After the response threshold was first crossed, four additional filaments were applied that varied sequentially up or down based on the animal response. The resulting pattern of positive and negative responses was tabulated and the 50% response threshold was calculated using the equation:

$$\text{50\% threshold (g)}=\frac{10^{\left[X_{f}+k\delta \right]}}{10,000}$$

where X_f_ represents the value of the final von Frey filament used, *k* represents the tabular value for the pattern of positive/negative responses (Dixon, 1980; Chaplan et al., 1994), and *δ* represents the mean difference (in log units) between stimuli.

### Measurement of fecal pellet output

One day after transduction, rats were placed individually in plastic cages (W, 375 mm × D, 480 mm × H, 210 mm) with free access to food and water. After a 4-h period, rats were removed, the bedding was carefully inspected, and the number of fecal pellets produced during this period was counted.

### Western blot analysis

Urinary bladders harvested from control and rats transduced with AdCldn2 were pinned in a square grid holder pad. Urothelial samples were obtained by gently scraping the epithelium into RIPA buffer [40 mM Tris, 150 mM NaCl, 2 mM EDTA, 10% (v/v) glycerol, 1% (v/v Triton X−100, 0.5% (w/v) sodium deoxycholate, 0.2% (w/v) SDS, and a Protease Inhibitor Cocktail Set III (EMD Bioscience); pH 7.6]. Extracts were rotated at 2000 rpm for 20 min at 4°C and then centrifuged at 25,000 × *g* for 20 min at 4°C. The supernatant was collected and placed in a −20°C freezer until further use. Spinal cord segments receiving bladder input [lumbosacral (LS; L6-S1) and thoracolumbar (TL; T13-L2)] were harvested from rats transduced with AdGFP or AdCldn2. Tissue was homogenized in RIPA buffer supplemented with protease inhibitor cocktail set III (EMD Bioscience) and phosphatase inhibitor cocktail (Cell Signaling Technology) in a disperser device (Polytron PT 10−35 GT, Kinematica) at 4°C. The homogenate was centrifuged at 25,000 × *g* for 20 min at 4°C. The supernatant was collected in a new tube and kept on ice for Western blot analysis. Protein concentration in the samples was determined with BCA Protein Assay (Thermo Fisher Scientific). Urothelial samples were mixed in a 1:1 ratio with loading buffer (Laemmli sample buffer supplemented with 0.277 M SDS and 1.420 M β-mercaptoethanol) and then incubated for 45 min at 37°C. Spinal cord samples were mixed in a 1:1 ratio with Laemmli sample loading buffer and heated at 95°C minutes for 5 min. Protein samples were loaded onto Criterion TGX 12% gels (Bio-Rad) and resolved by electrophoresis at 180 V for 50 min. Proteins were transferred to nitrocellulose membranes using a Trans-Blot Turbo Transfer system (Bio-Rad) according to the manufacturer’s instructions. Membranes were blocked with PBS supplemented with 10% (w/v) nonfat milk for 1 h. After blocking, membranes were incubated with a Cldn2 (1:5000), β-actin (1:5000), ERK (1:1000), or a pERK (1:2000) primary antibody overnight at 4°C. The next day, membranes were incubated with the corresponding secondary antibodies conjugated with peroxidase (1:5000; KPL). Bands were visualized using Western Lightning Chemiluminescence Reagent Plus (PerkinElmer) and quantified with ImageJ.

### c-Fos immunostaining in spinal cord segments

Rats were anesthetized with isoflurane (2–4% in O_2_) and urethane (1.2 g/kg; Sigma). For acetic acid positive controls, urinary bladders were catheterized through the dome using a flared PE50 catheter connected to an infusion pump. Acetic acid (1% in saline) was infused for 2 h at a rate of 0.1 ml/min, after which the rats were perfused through the heart with PBS and 4% (v/v) paraformaldehyde in PBS. Anesthetized rats transduced with AdGFP and AdCldn2 were perfused through the heart with PBS and then with by 4% (v/v) paraformaldehyde in PBS. Spinal cord segments (L4, L6-S1, T13-L2, and C4) were collected, post-fixed for ∼2 h in 4% (v/v) paraformaldehyde, and immersed in 20% sucrose in PBS for ∼24 h and then in 30% sucrose in PBS for 24–48 h. Tissue was embedded in a 50/50 mixture of O.C.T. and sucrose (30% w/v in PBS) and stored at −80°C. Frozen tissue blocks were sectioned using a CM1950 cryostat (Leica); 35-μm sections were immediately placed in 0.1 M sodium phosphate buffer, pH 7.2 (free floating sections). To block endogenous peroxidase activity, sections were treated with 0.15% (w/v) H_2_O_2_ in sodium phosphate buffer. After several rinses with sodium phosphate buffer, sections were incubated overnight with a rabbit antibody to c-Fos (1:5000; EnCor Biotechnology) in blocking buffer (sodium phosphate buffer supplemented with 1% BSA, 1% normal donkey serum and 0.3% Triton X-100). Sections were rinsed in sodium phosphate buffer and then incubated with a biotinylated donkey antibody to rabbit IgG (1:500) in blocking buffer for 1 h at room temperature. After additional rinses, the tissue was incubated in Avidin-Biotin Complex (VectaStain Elite Reagents, Vector Labs) in blocking buffer for 1.5 h at room temperature. After several rinses with sodium phosphate buffer, the tissue was treated with 0.1 M sodium acetate buffer, pH 4, for 10 min. For staining, sections were immersed in filtered diaminobenzadine solution containing 2.5% (w/v) NiSO_4_ and 0.003 % (w/v) H_2_O_2_ in sodium acetate buffer as previously described (Mantella et al., 2003; Rinaman, 2003). To stop the peroxidase catalyzed reaction, tissue was placed in 0.1 M sodium acetate buffer. After several washes in sodium phosphate buffer, sections were mounted onto charged glass microscopic slides (Superfrost Plus; Thermo Fisher Scientific) and left at room temperature overnight to dry. Tissue was dehydrated in a graded ethanol series and defatted in xylene. Slides were coverslipped using Cytoseal 60 (Thermo Fisher Scientific). Tile scanning of spinal cord sections was performed with a Leica TCS SP8 confocal microscope equipped with a 20× (Dry; N.A. = 0.75) objective, resonant scanner, and 638 laser line to generate transmitted light stacks with 10 μm step size. Automated merging was achieved with LASX acquisition software using smooth overlap blending. The absolute number of c-Fos-positive cells in spinal cord sections was quantified with ImageJ 1.51k (NIH) using the multi-point tool. For each spinal cord segment, the mean number of c-Fos-positive cells was estimated from three randomly selected spinal cord sections.

### Retrograde labeling of bladder sensory neurons

Bladder afferent neurons were labeled with the fluorescent dye DiI (1,1'-dioctadecyl-3,3,3',3'-Tetramethylindocarbocyanine perchlorate, Invitrogen) as reported in the literature (Yoshimura et al., 1996; Yoshimura and de Groat, 1999; Yoshimura et al., 2003; Dang et al., 2005; Dang et al., 2008). Briefly, rats were anesthetized with isoflurane and the bladder was exposed through an abdominal incision (∼1 cm in length). Dil (5% w/v in DMSO) was injected at four to six sites (total volume, 20–30 μl) in the bladder wall with a syringe. At each injection site, the needle was kept in place for 20–30 s after inoculation. Any visible leakage of dye was removed by application of a cotton swab and rinsed with saline. The muscle layer and skin incision were individually closed with 5.0 and 3.0 silk sutures (AD Surgical), respectively. Postoperative analgesia was provided by subcutaneous administration of ketoprofen (5 mg/kg, Zoetis). Ampicillin (10 mg/kg, Boehringer Ingelheim Vetmedica) was administrated to prevent infections. Rats were housed under the conditions described above between 8 and 12 d before any further procedure was performed.

### Isolation of bladder sensory neurons

Pelvic and hypogastric nerve bladder afferents have cell bodies located in LS L6-S2 and TL T13-L2 DRG, respectively. To isolate bladder sensory neurons, L6-S2 or T13-L2 DRGs were harvested and transfered to a cell culture dish containing neurobasal media (Neuro-A medium supplemented with 5% of B27 supplements, 0.5 mM L-glutamine, and 10 U/ml of penicillin/streptomycin mixture, Invitrogen). DRGs were minced and agitated in a cell culture flask containing 5 ml of neurobasal media supplemented with 10 mg of collagenase type 4 (Worthington Biochemical) and 5 mg of trypsin (Worthington Biochemical) for 30 min at 37°C. Tissue fragments were gently triturated with a fire-polished glass pipette and the cell suspension was centrifuged at 420 × *g* for 5 min. The pellet, containing DRG somas, was resuspended in neurobasal media. The centrifugation and resuspension steps were repeated three times. Finally, the pellet was resuspended in 1.5 ml of neurobasal media, and the suspension was plated on coverslips coated with ornithine and laminin inside a six-well tissue culture plate. After an incubation of 2 h at 37°C with 5% CO_2_, 3 ml of warm neurobasal media was added to each well and the tissue culture plate was returned to the incubator. Electrophysiological studies were performed within 2 and 10 h of plating.

### Patch-clamp studies

Whole-cell patch-clamp recordings from acutely dissociated bladder sensory neurons were obtained with the perforated patch technique using Amphotericin B. Current-clamp recordings were performed at room temperature with a PC-505B patch-clamp amplifier (Warner Instruments). Glass coverslips with DRG neurons were transferred to a chamber mounted on the stage of a Nikon Ti inverted microscope equipped a Sedat Quad set (Chroma Technology), a PhotoFluor II metal halide light source (89 North), a Lambda 10-3 filter wheel system (Sutter Instruments), and an ORCA-Flash 2.8 camera (Hamamatsu). Micropipettes were pulled from borosilicate glass capillary tubes (Warner Instruments) using a PP-81 puller (Narishige). Fire-polished micropipettes with a tip resistance of 1.5-3 mΩ were used for current-clamp recordings. The pipette filling solution contained: 145 mM KCl, 1 mM MgCl_2_, 0.1 mM CaCl_2_, 1 mM EGTA, and 10 mM HEPES; pH 7.2. Amphotericin B was added to the pipette solution to a final concentration of 120 μg/ml. The extracellular bath solution contained: 138 mM NaCl, 5 mM KCl, 0.5 mM MgCl_2_, 1.5 mM CaCl_2_, and 10 mM HEPES; pH 7.4. After establishing whole-cell configuration in voltage-clamp mode, the membrane potential was clamped at −60 mV and the cell capacitance was obtained by reading the value for input capacitance neutralization directly from the amplifier. To study firing and action potential properties, the amplifier was switched to current-clamp mode. Signals were low-pass filtered at 1 kHz (four-pole Bessel filter) and digitized with a Digidata 1440A (Molecular Devices) at 5 kHz. Command protocols, data acquisition and analysis were controlled by pClamp 10 software (Molecular Devices).

### Data analysis

Based on the presence or absence of spontaneous action potentials during a 5-min period immediately after achieving whole-cell configuration, sensory neurons were classified as silent or spontaneously active. The passive and active electrical properties of DRG neurons were determined in the current-clamp mode. To determine the input resistance, I/V relationships were generated by injecting 400-ms current steps from −75 to 50 pA in steps of 25 pA. The input resistance was calculated from the slope of I/V relationships. For silent neurons, only those that had a resting membrane potential more negative than −40 mV and generated action potentials with a distinct overshoot higher than 0 mV in response to depolarizing current injections were studied. The action potential properties of bladder sensory neurons were examined as previously described (Gold and Traub, 2004; Dang et al., 2008). A series of 4-ms rectangular depolarizing current pulses of increasing intensity were injected until an action potential was evoked (Fig. 5*C*). The following active electrical properties were measured for silent bladder sensory neurons: resting membrane potential (a), action potential overshoot above 0 mV (b), action potential duration at 0 mV (c), magnitude of hyperpolarization below the resting membrane potential (d), action potential threshold (e), and rheobase (f; Fig. 5*C*). Action potential rheobase and threshold are defined as the minimum depolarizing current injection necessary to evoke an action potential and the maximum membrane potential depolarization obtained in the absence of an action potential, respectively. To examine firing patterns in bladder sensory neurons, a series of 500-ms depolarizing rectangular current pulses equivalent to 1, 1.5, 2, 2.5, and 3 times the rheobase were injected every 4 s, and the voltage responses were recorded. To generate stimulus response relationships, the number of spikes evoked were counted and plotted against the corresponding injected current (1, 1.5, 2, 2.5, and 3 times rheobase).

### Statistical analysis

Data are expressed as mean ± SEM (*n*), where *n* equals the number of independent experiments. Parametric or nonparametric tests were employed as appropriate; *p* < 0.05 was considered statistically significant. Fitting and statistical comparisons were performed with Clampfit (Molecular Device), Sigmaplot 12.5 (Systat Software), or GraphPad 7 (GraphPad Software).

## Results

### Cldn2-induced cystitis

**Figure 1.**
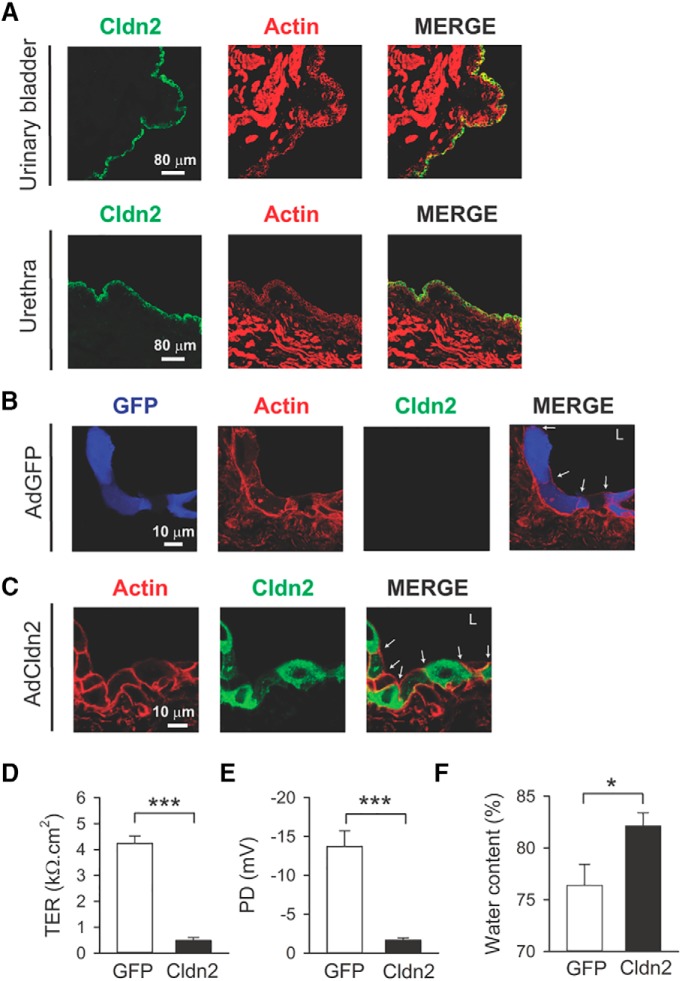
Overexpression of Cldn2 in the bladder urothelium induces cystitis. ***A***, Cross-sections of urinary bladder and urethra harvested from rats transduced with AdCldn2, fixed with paraformaldehyde, and stained with an antibody against Cldn2 (green) and Rhodamine Phalloidin (red). Note that the expression of Cldn2 is restricted to the umbrella cell layer in the urinary bladder and to the epithelium in the urethra. ***B***, ***C***, Cross-sections of bladders transduced with AdGFP (***A***) or AdCldn2 (***B***), fixed with paraformaldehyde, and stained with an antibody against Cldn2 (green) and Phalloidin Alexa Fluor 647 for actin (red). Arrows indicate TJs and L indicates lumen. ***D***, ***E***, Tissue electrical resistance (TER) and tissue electrical potential difference (PD) for urinary bladders transduced with AdGFP or AdCldn2. Urinary bladders from transduced rats were mounted in Ussing chambers and the electrical properties were measured as indicated in Materials and Methods. Statistically significant differences between experimental conditions are indicated as ***p < 0.001 (*n* = 6–9, Mann-Whitney nonparametric test). ***F***, Tissue water content of urinary bladders transduced with AdGFP or AdCldn2. A statistically significant difference between experimental conditions is indicated as *p < 0.05 (*n* = 6, Mann-Whitney nonparametric test).

Cldn2 is highly expressed in leaky epithelia such as the proximal tubule of the kidney and small intestine, but its expression is negligible in tight epithelia such as the distal nephron, colon, and urinary bladder (Zeissig et al., 2007; Angelow et al., 2008; Yu et al., 2009; Lameris et al., 2013; Montalbetti et al., 2015). Of significance, Cldn2 expression is upregulated in the distal colon of patient with inflammatory bowel disease and in the urinary bladder of patient with IC (Mankertz and Schulzke, 2007; Zeissig et al., 2007; Weber et al., 2008; Schulzke et al., 2009; Sanchez Freire et al., 2010). We previously showed that the overexpression of Cldn2 in the umbrella cell layer increases the permeability of the paracellular route toward cations, triggers an inflammatory process in the bladder mucosa and lamina propria with lymphocytic infiltration, and increases voiding frequency (Montalbetti et al., 2015). In the present study, to induce cystitis, rat urinary bladders were transduced with AdCldn2 using our established methods (Montalbetti et al., 2015). The urothelium endogenously expresses low amounts of Cldn2, which localizes along the lateral surfaces and at the TJ region of the umbrella cell layer (Montalbetti et al., 2015). As shown in Figure 1*A*,*C*, *in situ* transduction of bladders with AdCldn2 results in the overexpression of Cldn2 in the urothelium that lines the urinary bladder and urethra. The efficiency of transduction using this protocol was >95% (Fig. 1*A*). Note that the endogenous expression of Cldn2 in the urothelium is barely detectable under basal conditions (Montalbetti et al., 2015). In the current study, rats transduced with an AdGFP served as controls (Fig. 1*B*). Consistent with our previous studies (Montalbetti et al., 2015), the Tissue electrical resistance (TER) and tissue electrical potential difference (PD) of bladders transduced with AdCldn2 were significantly lower than those from rats transduced with AdGFP (Fig. 1*D*,*E*). In good agreement with our published histologic findings (Montalbetti et al., 2015), tissue water content, a measurement of edema, was significantly greater in bladders transduced with AdCldn2 than controls (GFP: 76.4 ± 2.0%, *n* = 6; Cldn2: 82.1 ± 1.3%, *n* = 6; *p* < 0.05; Fig. 1*F*).

### Overexpression of Cldn2 in the urothelium induces mechanical allodynia in the pelvic region

**Figure 2.**
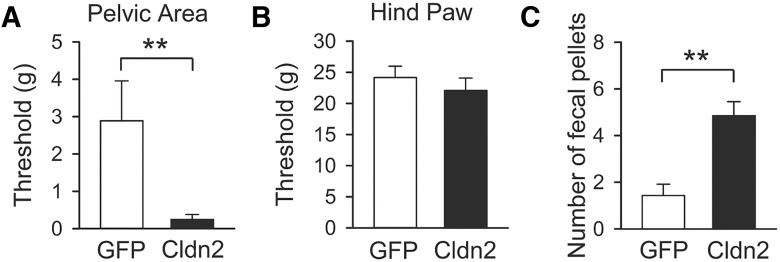
Overexpression of Cldn2 in the urothelium induces pelvic mechanical allodynia and organ cross-sensitization. ***A***, ***B***, Urothelial overexpression of Cldn2 reduces the threshold to mechanical stimuli applied to the pelvic area. Mechanical allodynia on the lower abdominal area close to the urinary bladder (***A***) and on the plantar surface of the hind paw (***B***) was evaluated with von Frey filaments in rats transduced with AdGFP or AdCldn2. 50% mechanical withdrawal threshold (g) was estimated with von Frey filaments applied in an up-down testing paradigm as previously described (Chaplan et al., 1994). Statistically significant difference between experimental conditions is indicated as **p < 0.01 (*n* = 7, Mann-Whitney nonparametric test). ***C***, Urothelial overexpression of Cldn2 sensitizes colon afferents. Number of fecal pellets in a 4 h period in rats transduced with AdGFP or AdCldn2. Statistically significant difference between experimental conditions is indicated as **p < 0.01 (*n* = 7, Mann-Whitney nonparametric test).

The presence of recurring discomfort and pain in the bladder and the surrounding pelvic region is a hallmark of IC/BPS. To determine whether the overexpression of Cldn2 in the urothelium alters somatic sensitivity in the pelvic area, we measured 50% mechanical withdrawal threshold (g) to von Frey filaments in rats transduced with AdCldn2 or AdGFP. Consistent with the presence of bladder-derived pain and an IC/BPS phenotype, rats transduced with AdCldn2 showed hypersensitivity (lower withdrawal threshold) to von Frey filaments applied to the pelvic region (Fig. 2*A*), but not to the hind paw (Fig. 2*B*). The 50% withdrawal threshold to mechanical stimuli applied to the pelvic region was 0.24 ± 0.13 g (*n* = 7) for rats transduced with AdCldn2 and 2.89 ± 1.07 g (*n* = 7, *p* < 0.01) for controls transduced with AdGFP. IC/BPS patients have a higher prevalence of irritable bowel symptoms than the general population (Alagiri et al., 1997; Howard, 2003; Buffington, 2004). To determine whether the overexpression of Cldn2 in the urothelium alters bowel function, we measured fecal pellet output as an index of intestinal motility (Barone et al., 1990). Significantly, the number of fecal pellets collected from rats transduced with AdCldn2 was larger than the number collected from rats transduced with AdGFP (Fig. 2*C*). Together, our results indicate that increased urothelial TJ permeability reduces the threshold to mechanical stimuli in the lower abdominal area and adversely alters bowel activity.

### Overexpression of Cldn2 in the urothelium activates nociceptive pathways in spinal cord segments receiving bladder input

**Figure 3.**
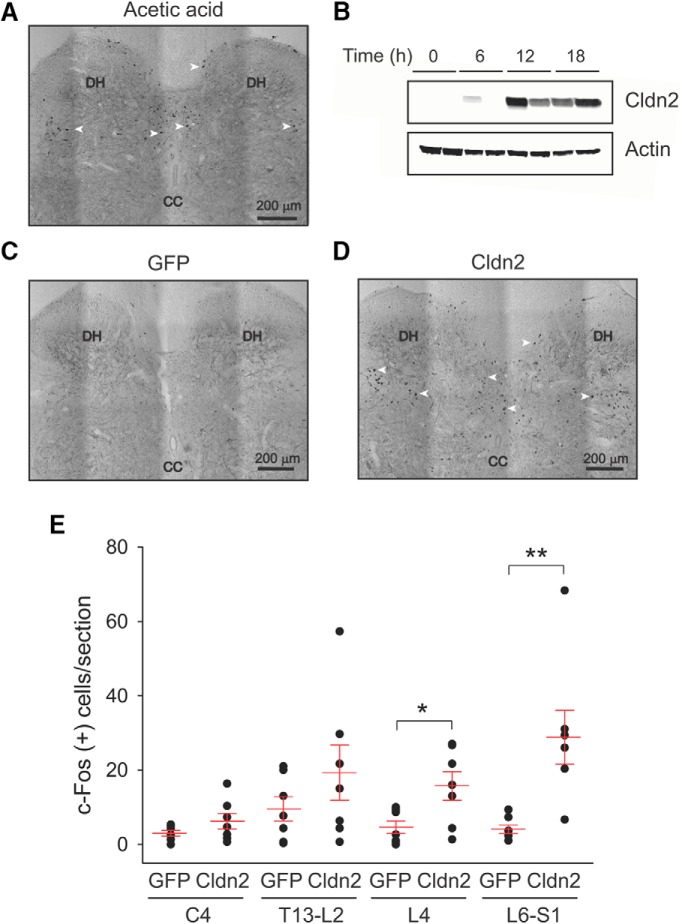
Overexpression of Cldn2 in the urothelium increases the number of c-Fos-immunoreactive cells in spinal cord segments receiving bladder input. ***A***, Micrography of an L6-S1 spinal cord section from a rat infused with 1% acetic acid for 2 h showing the distribution of c-Fos-positive neurons in the dorsal horn (DH). Immunoreactive neurons were distributed throughout the medial DH, dorsal commissure, and sacral parasympathetic nucleus. Arrows indicate areas with c-Fos-positive cells. ***B***, Time course of expression of Cldn2 following *in situ* transduction with AdCldn2. Urothelial samples were collected 6, 12, and 18 h after *in situ* transduction. Nontransduced rats served as controls for this experiment (time 0 h). Whole-cell lysates were subjected to immunoblot with an anti-Cldn2 antibody or an anti-actin antibody. ***C***, Micrography of an L6-S1 spinal cord section from a rat transduced with AdGFP. ***D***, Micrography of an L6-S1 spinal cord section from a rat transduced with AdCldn2. Arrows indicate areas with c-Fos-positive cells. c-Fos-positive cells were distributed throughout the medial DH, dorsal commissure, and sacral parasympathetic nucleus. ***E***, Number of c-Fos-positive neurons per section in spinal cord segments from rats transduced with AdGFP and AdCldn2. Statistically significant differences between experimental conditions are indicated as *p < 0.05 and **p < 0.01 (*n* = 7, Mann-Whitney nonparametric test).

To determine whether increased urothelial TJ permeability activates nociceptive pathways in the spinal cord, we examined the expression of c-Fos and activation of ERK1/2 (phosphorylation) in segments receiving bladder input from rats transduced with AdGFP or AdCldn2. Under physiologic conditions, c-Fos is not expressed in the spinal cord and ERK1/2 activity is low (Cruz and Cruz, 2007; Gao and Ji, 2009). Previous studies showed that noxious and mechanical stimulation of the lower urinary tract markedly increases c-Fos expression in dorsal horn neurons of the L6-S1 spinal cord (Birder and de Groat, 1992; Cruz et al., 1994; Vizzard, 2000a, 2000b,). To assess the specificity of the antibody and optimize the immunocytochemical reaction, we examined the expression of c-Fos in rats infused with 1% acetic acid in saline for 2 h. Consistent with previous studies, chemical irritation of the urinary bladder induced the expression of c-Fos in the posterior horn of the L6-S1 spinal cord (Fig. 3*A*), but not L4 (data not shown). c-Fos-positive cells were distributed as previously described throughout the medial dorsal horn, dorsal commissure and sacral parasympathetic nucleus (Birder and de Groat, 1992).

Following acute chemical irritation of the lower urinary tract with acetic acid, c-Fos expression in LS (L6-S1) spinal cord segments peaks 1–2 h after the initial expo-sure, begins to decline at 6 h and returns to basal levels 24 h after exposure (Birder and de Groat, 1992). If increased urothelial permeability causes noxious stimulation of bladder afferents, then c-Fos expression should increase along with the expression of Cldn2. To determine the time course of Cldn2 expression in the urothelium following *in situ* transduction, we performed Western blot analysis with urothelial samples collected 6, 12, and 18 h after transduction. Nontransduced rats serve as controls for this experiment (time 0). As shown in Figure 3*B*, the expression of Cldn2 reaches to a peak 12 h after transduction. Therefore, c-Fos expression in spinal cord segments receiving afferent input from the pelvic (L6-S1) and hypogastric (T13-L2) nerves was examined 12 h after transduction. Consistent with previous studies, c-Fos-positive cells were distributed in the dorsal horn areas that receive bladder afferent input: medial dorsal horn, dorsal commissure, and sacral parasympathetic nucleus (Fig. 3*D*). The number of c-Fos-positive cells in spinal cord segments receiving afferent input from the pelvic (L6-S1) nerve from rats transduced with AdCldn2 was significantly larger than in those transduced with AdGFP (Fig. 3*E*). No statistically significant differences in the number of c-Fos-positive cells in C4 and T13-L2 spinal cord segments were observed between rats transduced with AdGFP and AdCldn2. Unexpectedly, the number of c-Fos-positive cells in L4 segments was significantly larger in rats transduced with AdCldn2 than AdGFP (Fig. 3*E*). These results suggest that increased urothelial permeability activates nociceptive pathways in the spinal cord.

**Figure 4.**
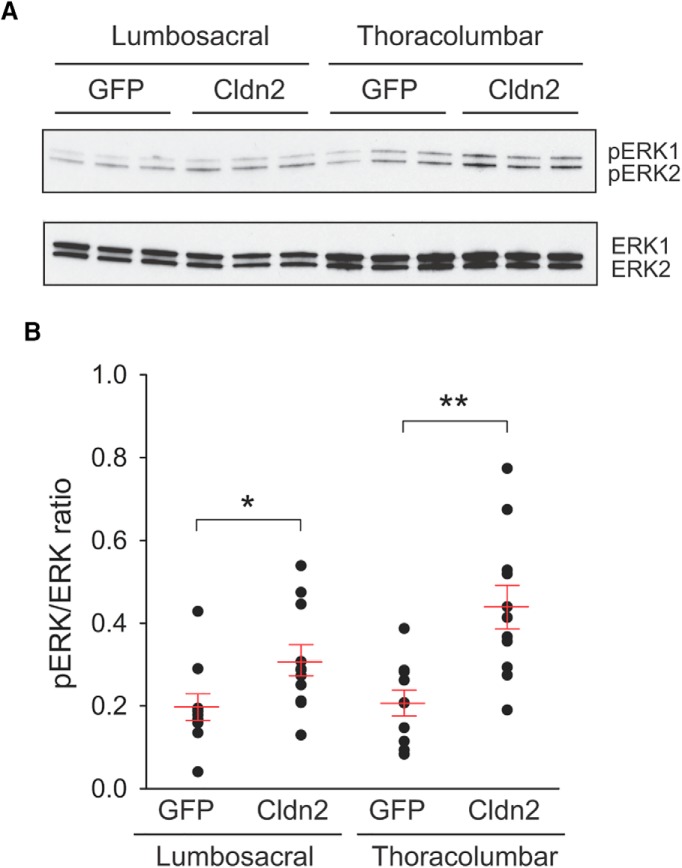
Overexpression of Cldn2 in the urothelium activates ERK in spinal cord segments receiving bladder input. ***A***, Representative Western blotting of total (ERK) and phosphorylated (pERK) forms of ERK1/2 in spinal cord segments receiving pelvic (LS) and hypogastric (TL) nerve afferents from rats transduced with AdGFP or AdCldn2. ***B***, Analysis of pERK/ERK ratio in LS and TL segments of rats transduced with AdGFP or AdCldn2. Statistically significant differences between experimental conditions are indicated as *p < 0.05 and **p < 0.01 (*n* = 10–11, Mann-Whitney nonparametric test).

ERK1/2 is rapidly activated (phosphorylated) in the spinal cord dorsal horn following noxious peripheral stimulation and its expression correlates well with pain behavior assessments in experimental animals (Kato et al., 1995; Sweatt, 2001; Dai et al., 2002; Ji et al., 2002; Obata et al., 2004; Corrow and Vizzard, 2009; Gao and Ji, 2009; Liu et al., 2011; Liu et al., 2012; Sadler et al., 2014; DeBerry et al., 2015). To determine whether increased urothelial permeability activates the ERK pathway in spinal cord segments receiving bladder afferent input, we performed Western blot analysis of total (ERK) and the phosphorylated forms of ERK1/2 (pERK) 24 h after transduction with AdGFP or AdCldn2 (Fig. 4). Consistent with the notion that increased urothelial TJ permeability activates nociceptive pathways, we observed a significant increase in pERK/ERK ratio in spinal cord segments receiving bladder input from the hypogastric (TL) and pelvic (LS) nerves of animals transduced with AdCldn2, when compared with controls (Fig. 4*B*). Taken as a whole, our studies show that reduced urothelial TJ barrier function promotes noxious stimulation of bladder afferent pathways.

### Overexpression of Cldn2 in the urothelium promotes spontaneous firing of bladder afferent neurons

**Figure 5.**
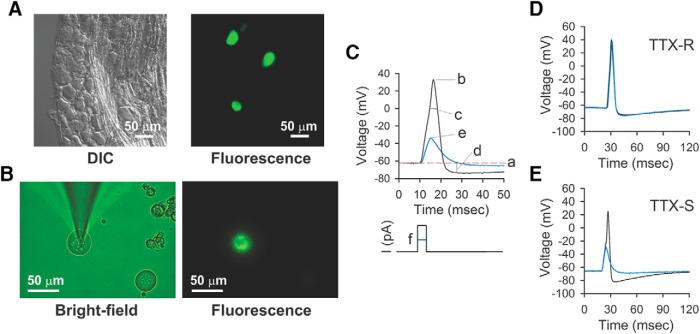
Analysis of electrical properties of bladder sensory neurons. DRGs were harvested from rats injected with DiI into the bladder wall. ***A***, DIC and epifluorescence micrographies captured from a cryosection of a paraformaldehyde fixed DRG (L1). ***B***, Bright field and epifluorescence micrographies of acutely isolated LS (L6-S2) DRG neurons captured during a patch-clamp experiment. ***C***, Analysis of passive and active action potential properties. Representative voltage trace registered in the current-clamp mode with the perforated patch-clamp technique from a Dil-labeled bladder sensory neuron. Action potentials were evoked by 4-ms depolarizing current pulses through the recording electrode. The current injection protocol is shown beneath voltage trace. Letters refer to properties of the action potential examined. a, resting membrane potential; b, action potential overshoot (above 0 mV); c, action potential duration at 0 mV; d, magnitude of after hyperpolarization (AHP) below resting membrane potential (in mV); e, action potential threshold, which is defined as the greatest membrane potential (mV) achieved in response to a current pulse that does not trigger an action potential; f, rheobase, which is defined as the smallest amount of depolarizing current (pA) required to trigger an action potential. ***D***, ***E***, Classification of DiI-labeled bladder neurons on the basis of the sensitivity of the action potential to TTX. Representative tracing of DiI-labeled bladder neurons with TTX-R (***D***) and TTX-S (***E***) action potentials.

**Figure 6.**
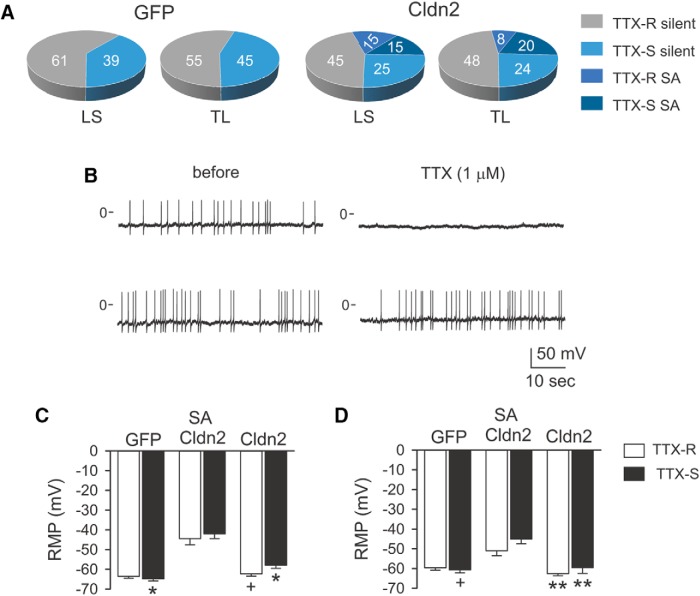
Overexpression of Cldn2 in the urothelium induces spontaneous activity in both LS and TL bladder sensory neurons. LS (L6-S2) and TL (T13-L2) DRGs were harvested from rats 9–13 d after injection of DiI in the bladder wall. The day before DRG collection, rats were transduced with AdGFP or AdCldn2. DRG neurons were isolated and cultured as indicated in Materials and Methods. Action potential activity in acutely isolated bladder sensory neurons was examined with the perforated patch-clamp technique. Sensory neurons were classified on the basis of their origin (LS or TL) and sensitivity of the action potential to 1 μM TTX (TTX-S or TTX-R). ***A***, Proportion of silent and spontaneously active (SA) LS and TL neurons from rats transduced with AdGFP or AdCldn2. Neither LS nor TL bladder DRG neurons were spontaneously active in rats transduced with GFP (0/112). Approximately 30% (LS, 20/67 and TL, 15/54) of the neurons harvested from rats transduced with AdCldn2 showed spontaneous activity. Of the bladder neurons with spontaneous activity harvested from rats transduced with AdCldn2, 15% (10/67) of the LS sensory neurons exhibited TTX-S action potentials, while 20% (11/54) of the TL sensory neurons exhibited TTX-S action potentials. Data were collected from 12 rats transduced with AdGFP and 12 rats transduced with AdCldn2. ***B***, Representative voltage tracings from bladder sensory neurons harvested from rats transduced with AdCldn2 with spontaneous TTX-S (upper panel) or TTX-R (lower panel) action potentials. ***C***, Resting membrane potential of LS bladder sensory neurons. Statistically significant differences between neurons with spontaneous action potentials and their TTX-S or TTX-R silent counterparts are indicated as *p < 0.05 and +*p* < 0.001 (*n* = 10–36, Kruskal-Wallis test followed Dunn's multiple comparisons test). ***D***, Resting membrane potential of TL bladder sensory neurons. Statistically significant differences between neurons with spontaneous action potentials and their TTX-S or TTX-R silent counterparts are indicated as **p < 0.01 and +*p* < 0.001 (*n* = 4–29, Kruskal-Wallis test followed Dunn's multiple comparisons test).

To determine whether the mechanical allodynia observed in rats overexpressing Cldn2 in the urothelium results from altered afferent activity, we conducted patch-clamp studies with acutely isolated bladder sensory neurons harvested from rats transduced with AdGFP or AdCldn2. To label bladder afferents, DiI was injected into the bladder wall. The dye is transported in a retrograde fashion and reaches somas in LS (L6-S2) and TL (T13-L2) DRG one to two weeks after injection. Figure 5*A* shows DIC and epifluorescence micrographs from a lumbar 1 (L1) DRG harvested from a rat injected with DiI into the bladder wall. Bright-field and epifluorescence micrographs of acutely isolated LS (L6-S2) DRG neurons from a rat injected with DiI are shown in Figure 5*B*. The activity and electrical properties of acutely isolated bladder sensory neurons were examined with the perforated patch-clamp technique in the current-clamp mode (Fig. 5*C*). The amphotericin B-perforated patch-clamp technique has the advantage of maintaining the cytosolic composition without altering endogenous levels of Ca^2+^ and signaling molecules. Previous patch-clamp studies performed with retrogradely labeled bladder neurons described two general populations, one with tetrodotoxin-resistant (TTX-R) action potentials and another with TTX-sensitive (TTX-S) action potentials (Yoshimura et al., 1996). The population of neurons with TTX-R action potentials is sensitive to capsaicin and is likely to be of C-fiber origin (Yoshimura and de Groat, 1999). Most Aδ fiber afferent neurons exhibit TTX-S action potentials and are insensitive to capsaicin (de Groat and Yoshimura, 2009). In the present study, we classified the action potentials of bladder sensory neurons as TTX-S or TTX-R. Representative recordings of bladder sensory neurons with TTX-S and TTX-R action potentials are shown in Figure 5*D*,*E*. In controls (AdGFP-transduced) rats, a similar proportion of LS (61%, 36/59) and TL (55%, 29/53) bladder sensory neurons exhibited TTX-R action potentials (Fig. 6*A*). The whole-cell membrane capacitance of the neurons harvested from rats transduced with AdGFP with TTX-R action potentials was similar to the neurons with TTX-S action potentials (Tables 1, 2). Whereas bladder afferent neurons harvested from rats transduced with AdGFP were electrically silent (LS, 0/59; TL, 0/53), ∼30% (LS, 20/67; TL, 15/54) of the bladder afferents from rats transduced with AdCldn2 exhibited spontaneous action potentials (Fig. 6*A*). Of the LS bladder sensory neurons with spontaneous activity, 50% (10/20) exhibited TTX-S action potentials (Fig. 6*A*). Among the TL bladder sensory neurons with spontaneous activity, the majority exhibited TTX-S action potentials (11 of 15). The whole-cell membrane capacitance of bladder sensory neurons harvested from rats transduced with AdCldn2 with spontaneous activity (32.8 ± 2.1 pF, *n* = 33) did not differ from that of silent neurons (30.0 ± 1.1 pF, *n* = 85, *p* = 0.24, unpaired *t* test with Welch’s correction). For the most part, whole-cell membrane capacitance and input resistance of silent bladder sensory neurons harvested from rats transduced with GFP were of similar magnitude to those from neurons harvested from rats transduced with Cldn2 (Tables 1, 2). In contrast to the overall increase in cell size reported in sensory neurons from rats treated with cyclosphosphamide (Yoshimura and de Groat, 1999; Dang et al., 2013), our results indicate that increased urothelial TJ barrier permeability does not alter the cell size distribution of the bladder sensory neurons. Spontaneously active neurons exhibited lower resting membrane potentials than silent neurons and irregular membrane potential oscillations (Fig. 6*C*,*D*). The membrane potential mean and oscillation amplitude for the LS bladder sensory neurons with spontaneous activity were −43.2 ± 1.9 mV (Fig. 6*C*) and 9.4 ± 0.7 mV (data not shown), and for the TL bladder sensory neurons with spontaneous activity were −46.7 ± 2.0 mV (Fig. 6*D*) and 9.8 ± 1.0 mV (data not shown), respectively.

**Table 1.** Passive and active electrical properties of LS bladder sensory neurons from rats transduced with AdGFP or AdCldn2

	GFP		Cldn2	
	TTX-S	TTX-R	TTX-S	TTX-R
Number of cells RMP (mV)	*n* = 23 −64.8 ± 1.1	*n* = 36 −63.6 ± 0.9	*n* = 18 −57.9 ± 1.6**	*n* = 29 −62.1 ± 1.1
C_m_ (pF)	30.8 ± 2.6	29.7 ± 1.8	30.1 ± 2.5	29.1 ± 1.3
R_In_ (GΩ)	0.88 ± 0.08	1.15 ± 0.09	0.83 ± 0.10	1.04 ± 0.08
AP threshold (mV)	−29.1 ± 0.7	−22.2 ± 0.5	−34.2 ± 0.7***	−23.8 ± 0.8
AP duration (ms)	3.5 ± 0.2	6.3 ± 0.4	4.1 ± 0.5	5.9 ± 0.7
AP overshoot (mV)	32.0 ± 2.8	38.0 ± 2.7	30.5 ± 3.7	40.0 ± 2.5
Rheobase (pA)	300 ± 45	393 ± 34	159 ± 38*	361 ± 45
AHP mag (mV)	−10.8 ± 1.2	−10.1 ± 0.8	−12.1 ± 1.6	−10.3 ± 0.6

Values are means ± SEM. Statistically significant differences compared with control (GFP) are indicated as *p < 0.05, **p < 0.01, and ***p < 0.001 (unpaired *t* test with Welch’s correction). RMP, resting membrane potential; C_m_, membrane capacitance; R_In_, input resistance; AP, action potential; AHP mag, magnitude of hyperpolarization below the resting membrane potential.

**Table 2.** Passive and active electrical properties of TL bladder sensory neurons from rats transduced with AdGFP or AdCldn2

	GFP		Cldn2	
	TTX-S	TTX-R	TTX-S	TTX-R
Number of cells RMP (mV)	*n* = 24 −60.6 ± 1.6	*n* = 29 −59.6 ± 1.2	*n* = 13 −59.5 ± 2.9	*n* = 26 −62.5 ± 1.2
C_m_ (pF)	35.3 ± 2.7	32.5 ± 1.9	37.2 ± 3.8	26.7 ± 2.1*
R_In_ (GΩ)	0.65 ± 0.10	0.81 ± 0.10	0.67 ± 0.08	1.00 ± 0.09
AP threshold (mV)	−26.6 ± 0.8	−20.4 ± 0.9	−31.3 ± 0.7***	−21.1 ± 0.5
AP duration (ms)	3.3 ± 0.3	4.3 ± 0.3	3.6 ± 0.4	3.6 ± 0.3
AP overshoot (mV)	34.5 ± 2.8	33.1 ± 2.3	24.2 ± 2.5**	35.0 ± 1.9
Rheobase (pA)	500 ± 90	393 ± 55	384 ± 82	511 ± 52
AHP mag (mV)	−14.3 ± 1.6	−13.5 ± 1.6	−11.6 ± 1.4	−13.6 ± 2.1

Values are means ± SEM. Statistically significant differences compared with control (GFP) are indicated as *p < 0.05, **p < 0.01, and ***p < 0.001 (unpaired *t* test with Welch’s correction). RMP, resting membrane potential; C_m_, membrane capacitance; R_In_, input resistance; AP, action potential; AHP mag, magnitude of hyperpolarization below the resting membrane potential.

### Overexpression of Cldn2 in the urothelium sensitizes TTX-S bladder sensory neurons

To assess whether the overexpression of Cldn2 in the urothelium alters the excitability of bladder afferents, we examined the passive and active electrical properties of silent bladder sensory neurons harvested from rats transduced with AdGFP or AdCldn2. Consistent with previous studies (Yoshimura et al., 1996), bladder afferent neurons with TTX-R action potentials harvested from rats transduced with AdGFP (control) exhibited action potentials with higher threshold than the counterparts with TTX-S action potentials (Tables 1, 2). The overexpression of Cldn2 in the urothelium did not change the active or passive membrane properties of LS or TL bladder sensory neurons with TTX-R action potentials (Tables 1, 2). However, LS neurons with TTX-S action potentials from rats transduced with AdCldn2 have lower resting membrane potential, action potential threshold and rheobase than counterparts from rats transduced with AdGFP (Table 1). Similarly, we observed lower action potential threshold and action potential overshoot in TL sensory neurons with TTX-S action potentials harvested from rats transduced with AdCldn2 than control counterparts (Table 2). Taken together, our results indicate that increased urothelial TJ barrier permeability alters the passive and active electrical properties of the bladder sensory neurons with TTX-S action potentials. Because hypogastric and pelvic afferents mediate divergent functions (Dang et al., 2008), it is not surprising that increased urothelial TJ barrier permeability affects different passive and active electrical properties in LS and TL bladder sensory neurons.

**Figure 7.**
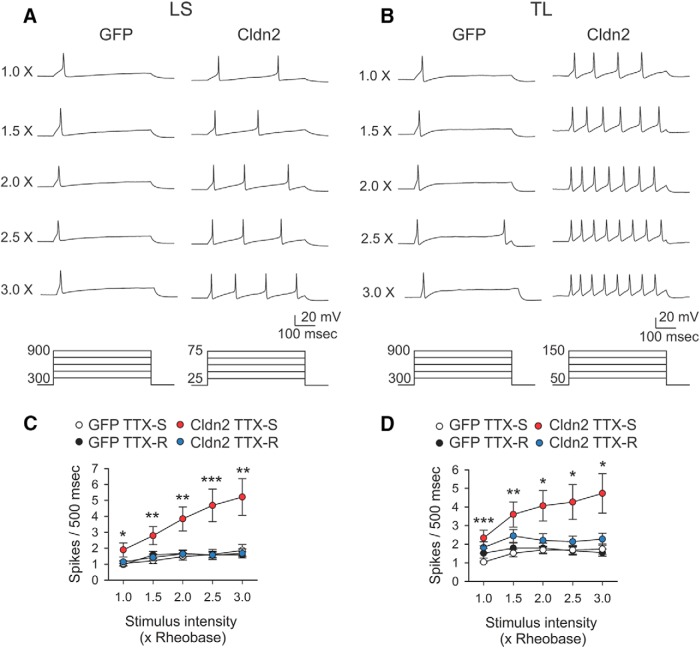
Overexpression of Cldn2 in the urothelium sensitizes LS and TL bladder sensory neurons with TTX-S action potentials. LS (L6-S2) and TL (T13-L2) DRGs were harvested from rats transduced with AdGFP or AdCldn2. The response of sensory neurons to suprathreshold stimulation was examined with the perforated patch-clamp technique. ***A***, ***B***, Representative tracings of action potential firing pattern in response to suprathreshold stimulation for LS (***A***) and TL (***B***) bladder DRG neurons with TTX-S action potentials. Action potentials were evoked by the injection of 500-ms depolarizing current pulses [1, 1.5, 2, 2.5, and 3 times (X) the rheobase] through the patch pipette. Current pulse protocols are shown at the bottom of the panels. ***C***, ***D***, Stimulus response relationships for LS (***C***) and TL (***D***) bladder sensory neurons. Action potentials were evoked by the injection of depolarizing current pulses as indicated above. The number of spikes evoked in response to stimuli of increased intensity for each neuron were computed. Sensory neurons were classified on the basis of their origin and the sensitivity of the action potential to 1 μM TTX. Statistically significant differences between neurons with TTX-S action potentials from rats transduced with AdCldn2 and control TTX-S counterparts are indicated as, *p < 0.05, **p < 0.01, and ***p < 0.001 (*n* = 15–36, Mann-Whitney nonparametric test).

Finally, to examine the excitability of LS bladder sensory neurons in response to electrical stimulation, we injected suprathreshold current pulses equivalent to 1, 1.5, 2, 2.5, and 3 times the rheobase for 500 ms with 4-s intervals. Consistent with previous studies (Yoshimura and de Groat, 1999; Yoshimura et al., 2003; Dang et al., 2008), sensory neurons harvested from rats transduced with AdGFP exhibited a phasic pattern of action potential firing in response to suprathreshold stimuli (Fig. 7*A*,*B*). No significant differences in the firing were observed among TTX-R neurons harvested from rats transduced with AdCldn2 or AdGFP (Fig. 7*C*, LS, *D*, TL). However, suprathreshold stimuli evoked a significantly greater number of spikes in bladder sensory neurons with TTX-S action potentials from rats transduced with AdCldn2 than in control counterparts (Fig. 7). Taken together, our studies indicate that increased TJ permeability sensitizes bladder sensory neurons with TTX-S action potentials, and that this process contributes to the bladder hyperreflexia and pelvic pain observed in rats transduced with AdCldn2.

## Discussion

Urothelial barrier function depends on the presence of high resistance TJs (Khandelwal et al., 2009), which not only regulate paracellular permeability, but may also play a role in the sensory function of the urothelium (Carattino et al., 2013). despite the prevalence of IC/BPS (>4,000,000 affected individuals in the United States), its etiology remains unknown. Animal models have provided limited insight into the pathophysiology of this disease, particularly with regard to the mechanisms that drive pelvic pain in IC/BPS patients. Most of the models of IC/BPS test the impact of extrinsic factors and insults but do not explore intrinsic defects that are known to be associated with IC/BPS (e.g., increased expression of Cldn2 message). Although irritants and immune stimulants increase urinary frequency, they fail to reproduce the localized abdominal pain present in IC/BPS patients (Lai et al., 2014). For instance, mice treated with the alkylating agent cyclophosphamide present generalized increased sensitivity to mechanical stimuli (Studeny et al., 2008; May and Vizzard, 2010). Likewise, models of experimental autoimmune cystitis exhibit increased pain response to noxious stimulation in the pelvic area as well as the hind paw (Bicer et al., 2015). Consistent with the presence of bladder-derived pain, rats overexpressing Cldn2 in the urothelium showed hypersensitivity to von Frey filaments applied to the pelvic region, but not to the hind paw. The reduction of the withdrawal threshold to von Frey filaments observed in rats transduced with AdCldn2 is more likely the result of noxious stimulation of the afferent pathways. In support of this notion, we noticed an increase in the expression of c-Fos and activation of ERK1/2 in spinal cord segments receiving bladder input. Unexpectedly, c-Fos expression was increased in L4 segments of rats transduced with AdCldn2 when compared with controls. Since bladder afferents from the urinary bladder project to discrete regions of the L6-S1 and T13-L2 spinal cord (Yoshimura and Chancellor, 2003; de Groat and Yoshimura, 2009; de Groat and Wickens, 2013), we posit that the observed increase in the expression of c-Fos in L4 segments of rats transduced with AdCldn2 results from the activation of ascending pathways, or from biting/scratching behavior as a response to visceral pain and irritation. Together, our studies indicate that the model of cystitis induced by the overexpression of Cldn2 in the urothelium resembles the symptoms and histologic features of human IC/BPS, particularly the bladder-derived pelvic pain.

Sustained increase in spontaneous afferent activity has a well-established role in ongoing pain (Djouhri et al., 2006; Yang et al., 2014) and has been shown to be one of the immediate events after peripheral nerve damage (Burchiel et al., 1985; Study and Kral, 1996; Xie et al., 2005), spinal cord injury (Bedi et al., 2010; Yang et al., 2014; Bavencoffe et al., 2016), and tissue inflammation (Koltzenburg et al., 1999; Djouhri et al., 2006; Xiao and Bennett, 2007). Spontaneous activity of primary sensory neurons can induce and maintain central sensitization (Ji and Woolf, 2001; Xie et al., 2005), while pharmacological blockade of spontaneous afferent activity immediately after peripheral nerve injury has been shown to permanently reduce or eliminate spontaneous pain, thermal and mechanical hyperalgesia (Xiao and Bennett, 1995; Lyu et al., 2000; Chaplan et al., 2003; Xie et al., 2005). Dang and colleagues reported that 35% of the LS and 45% of the TL bladder sensory neurons from rats treated chronically with cyclophosphamide, but not controls, exhibit spontaneous activity (Dang et al., 2008). Our studies shown that ∼30% of LS and TL bladder sensory neurons harvested from rats transduced with AdCldn2, but not with AdGFP, exhibit spontaneous activity. We found that bladder sensory neurons with TTX-S action potentials harvested from animals transduced with AdCldn2 exhibited increased response to suprathreshold stimulation as well as marked differences with respect to the passive and active electrical properties, when compared with neurons harvested from rats transduced with AdGFP. Together, these results support the notion that in the face of a leaky urothelium, spontaneous activity and sensitization of afferents causes bladder hyperreflexia and drives pelvic pain.

The urine contains large amounts of small solutes, such as K^+^ (120–475 mM) and NH_4_^+^ (10.4–56.2 mM; Shevock et al., 1993), that can potentially permeate through the TJs and alter neuronal excitability and activity. Because overexpression of Cldn2 increases the permeability of the urothelium to small ions, without altering the barrier to large organic molecules (Montalbetti et al., 2015), we posit that afferent sensitization is prompted by urinary solutes that diffuse through TJs and accumulate in the bladder interstitium. Since even small changes in extracellular [K^+^] can exert profound effects on neuronal excitability, smooth muscle function, and inflammatory cell activation (Hodgkin and Horowicz, 1959; Czéh et al., 1981; Sykova, 1981; Renaud and Light, 1992; Overgaard et al., 1999; Levite et al., 2000; Matkó, 2003; Allen et al., 2008; Medford-Davis and Rafique, 2014), we speculate that urinary K^+^ contributes to the bladder hyperreflexia and pain observed in rats transduced with Cldn2. Although the overexpression of Cldn2 in umbrella cells does not affect the structural integrity of the umbrella cell layer, it generates an inflammatory response in the urinary bladder characterized by the presence of a lymphocytic infiltrate and edema (Montalbetti et al., 2015). Consequently, we cannot rule out that the inflammatory process triggered by the overexpression of Cldn2 in the urothelium contributes to some degree to the sensitization of bladder afferents.

The general consensus is that normal micturition depends on mechanosensitive Aδ fiber afferents that respond to bladder distention in the physiologic range (Fowler et al., 2008; de Groat and Yoshimura, 2009). The role of C fibers on bladder function is less clear. Initial reports indicate that bladder C fibers have high thresholds and respond to bladder distention only at elevated pressure (Jänig and Morrison, 1986; Bahns et al., 1987; Häbler et al., 1993). However, this notion was challenged by reports describing a subpopulation of bladder C fiber afferents that respond to bladder distention in the physiologic range of pressures (Floyd et al., 1976; Bahns et al., 1986; Sengupta and Gebhart, 1994; Wen and Morrison, 1995; Dmitrieva and McMahon, 1996). As with other afferents innervating hollow organ structures, a key feature of bladder afferent neurons is that they become sensitized after organ insult and in chronic pathologic conditions such as tissue inflammation or irritation (Dang et al., 2008; Dang et al., 2013; Yoshimura et al., 2014). Previous studies showed that chronic cyclophosphamide administration sensitizes mainly fibers of C origin (Maggi et al., 1992; Yoshimura and de Groat, 1999; Yoshimura et al., 2002; Hayashi et al., 2009). In contrast, our studies indicate that the overexpression of Cldn2 in the urothelium increases the excitability of bladder afferent with TTX-S action potential, which are considered of Aδ origin (Yoshimura and de Groat, 1999). We posit that this discrepancy reflects differences in the underlying mechanisms that lead to cystitis in rats treated with cyclophosphamide and those overexpressing Cldn2. We cannot rule out that during the course of experimental cystitis different afferent pathways are sensitized at early and late time points.

Several lines of research support the notion that Aδ bladder afferents contribute to lower urinary tract symptoms and pain in IC/BPS patients and cats with feline IC, a naturally occurring form of IC that presents with reduced urothelial barrier function. For instance, Aδ bladder afferents from cats with feline IC show greater sensitivity to pressure than those from normal cats (Roppolo et al., 2005). Moreover, clinical studies showed that capsaicin and resiniferatoxin, two substances that target C fibers, are ineffective in the treatment of IC/BPS symptoms (Guo et al., 2013; Foster and Lake, 2014). The present results indicate that increased urothelial TJ permeability augments the electrical excitability of bladder sensory neurons primarily of Aδ origin, which promotes voiding at low filling volumes and lowers pelvic pain threshold to mechanical stimuli. In summary, our manuscript presents critical new insights into the mechanisms by which urothelial TJ barrier dysfunction causes bladder hyperreflexia and pain, providing a rational foundation to treat IC/BPS.

## Synthesis

Reviewing Editor: Kirill Martemyanov, The Scripps Research Institute Decisions are customarily a result of the Reviewing Editor and the peer reviewers coming together and discussing their recommendations until a consensus is reached. When revisions are invited, a fact-based synthesis statement explaining their decision and outlining what is needed to prepare a revision will be listed below.

Synthesis.

Both reviewers agreed that results presented in the manuscript are potentially important and are of interest to neuro-urology field and understanding mechanisms of visceral pain initiation. The molecular link established in this study was deemed important and in general the reviewers were satisfied with the responses to their initial comments. However, the reviewers also identified several areas of lingering concerns and it was felt that these issues need to be addressed to increase confidence in the interpretation of the results and enhance impact of the report.

Specifically I would like to direct your attention to the following areas that will be critical for you to address upon revision of your manuscript.

1. The common area of concern identified by both reviewers pertains to the pERK studies. As reviewers suggest, ERK phosphorylation needs to be studied at nearby spinal sites as a control. The low levels of basal phosphorylation warrants exploring other antibodies for IHC and/or consider applying noxious stimulation (See references Ji et al, 2002 Nature Neuroscience; Cruz et al 2005 Eur J. Neuroscience for guidance) to increase the levels. Perhaps more representative blots could be chosen and the data need to be shown as a scatter-plot. Please refer to unedited comments below for additional details.

2. It would be very helpful to provide experimental evidence for the relevance of Cldn2 expression changes to pathological conditions. Please consider examining changes in Cldn2 expression either in BPS/IC patient tissues or relevant animal models of the disease.

3. It would be important to demonstrate the extent of the claudin over-expression across the bladder and proximal urethra. This would also benefit researchers who wish to use this manipulation in the future, especially to check that their over-expression is comparable. Please provide additional images to demonstrate this - e.g. lower magnification images that show a larger area of urothelium. Your response letter claims to have provided this but the reviewers find this not to be the case reporting that instead you provided a slightly larger image at a very similar magnification to the others, so the reader still has no further information to assess how consistently or broadly across the bladder surface the urothelium expression levels were altered. This should be a very easy image (or set of images) to generate.

4. Please elaborate the description on urethra clip procedure along the lines stated in the rebuttal letter to ensure compliance with ethical treatment of animal subjects.

5. It would be important to incorporate suggestions provided by reviewer #2 and address the concerns of this reviewer as detailed below.

For your reference, I am providing unedited comments provided by both reviewers below. I hope you find them helpful for improving your manuscript.

Specific Reviewer's Comments

Reviewer #1

This is an interesting study that adds to a complex area of research. Indeed, after so many years of clinical and fundamental research, the full pathophysiological mechanisms of BPS/IC remain to be fully elucidated. This study shows that by upregulating the expression of a single junctional protein rats present signs of pain and increased neuronal firing, two features present in both animal models of disease and BPS/IC patients. Although the authors refer that Cldn2 is upregulated in patients (and they refer to a clinical study), the truth is that study only demonstrated increased mRNA and not the protein, which do not always go hand in hand. Although the models of BPS/IC are not ideal and have a strong inflammatory component, I would suggest the authors to test the expression of Cldn2 in pathological conditions and, ideally, in bladder biopsies. As is, we do not know if the genetically driven Cldn2 overexpression is comparable to what is seen in pathological conditions.

Another point relates to pERK. The bands shown are barely visible and were only demonstrated in thoracic regions. In addition, as far as I understood, these are basal levels of activation. I wonder how much higher pERK levels induced by stimulation (eg, bladder stimulation) would be in transduced animals. I also missed immunohistochemistry analysis of spinal samples as pERK posive cells could be glial or neuronal cells, the location of which could having different meanings (eg, laminae I-I versus laminae IX and X). In addition, I would like to see bands also from the lumbar region (L5-L6) and, as a control, from high thoracic or cervical cord.

Reviewer #2

The authors have provided extensive discussion of the issues raised. While some issues were addressed satisfactorily in the rebuttal and revised manuscript, I was unable to find changes in areas directly related to several of my comments. My comments here are numbered to match those in the authors' rebuttal.

1.

a. Although the additional experiments could be challenging (e.g., requiring pharmacological or molecular modification of the inflammatory response), I do not agree that “there is no means to determine the contribution of the inflammation to the observed neural changes”. If additional experiments are not conducted, I suggest revising the relevant section of the Discussion to expand upon this current limitation, such as describing how this may be addressed in the future and how mechanistic hypotheses could look, if this causal relationship does or does not exist.

b. To clarify, I did not state or intend to infer that “any type of inflammatory processes impact bladder afferents in a similar manner”.

2. The additional image provided (new panel A) has very little difference in magnification than others in the original panel. The image is larger, but the calibration bars and the cell/nuclear sizes in A show that the magnification is very similar to the other panels. As such, the image provides only slightly more information than the other panels, providing a view of the suburothelial expression level in a very small region of bladder. My original suggestion to demonstrate the expression at lower magnification would have had the benefit of showing how consistent or otherwise the urothelium was transfected around the circumference of a bladder section and in different regions of the lower urinary tract (e.g. if the bladder neck and proximal urethra were also transfected). This information has still not been provided, in images or in the text; I was also unable to find this information in the 2015 AJP paper.

3. It is possible that the authors have misunderstood the meaning of my comment that referred to the difficulty in (ie limitations of) using the results from longer treatment periods (as published by other groups, for different cystitis models) to interpret the results from this 24h treatment period. I did not question the choice of experimental time point. I also did not suggest extrapolating from their data to published data from CYP studies. Quite the opposite - I still consider that the Discussion would benefit from making it clearer to the reader what limitations there may be in making inferences from studies with quite different treatment periods. Many of these studies are referred to in the Discussion. My reference to Dang et al was simply an example of a study that was cited but with a different post-treatment period.

4. There was no response to my proposal of strengthening the experiment by measuring ERK phosphorylation at other nearby spinal levels. It is useful to know that attempts were made to visualize ERK phosphorylation in spinal cords from these treated animals. It is surprising that they were unable to visualise ERK phosphorylation in rat cord, with either stimulus. I do not consider this an essential experiment but it would have greatly enhanced the work, especially because of the inherent limitations of Western blotting and its quantitation. The faint increase in signal from samples 1 and 3 is just visible but, if this is the best example from the 10-11 replicates, is still not impressive and raises the question of what was different about sample 2 (an animal more sensitive to the bladder manipulation perhaps?). Given this level of variability, it may be worthwhile to provide the ERK data as a scatter plot as well as a histogram.

5. The absence of published data to support a change in nerve density (increase due to collateral growth or decrease due to degeneration) is not surprising as, to my knowledge, very few groups (if any) have quantified nerve density at 24 hours after any bladder treatment. I am not aware of this type of anatomical measurement having been performed at this early time point or across bladder inflammation models. Also, as the authors have rightly pointed out, not all bladder inflammation models should be considered as the same, so even if this data did exist for a different model, this would not necessarily infer an absence of effect in this Claudin manipulation study. I don't see how the authors can be so confident that there are no changes, given the known rapid ability for plasticity and remodelling of peripheral terminals.

6. It is pleasing to learn that animals were randomized to blinded treatment and control groups. This should be stated in the Methods. It remains disappointing that estrus cycle stage was not monitored at the time of tissue removal and hope this simple assay is conducted in future studies.

7. The additional information re the method of urethra clipping is useful and reassuring. This additional information should be included in the manuscript.

8. The authors are correct - the term “retraction” rather than “contraction” was used. I apologise for this error, but my question is not altered by this - the method is not explained and “retraction” is no clearer. A common assay for assessing visceral pain in distensible organs (eg bladder, rectum) is the visceromotor reflex (VMR), which measures contraction of specific muscle groups. If this is the assay performed, it should be stated and described as such. If the authors are using a different assay (eg of different muscles or if their measurement is simply macroscopic visual assessment rather than measuring muscle activity), this should be explained - ie which particular muscles and the nature of the measurement.

9. I apologise that I missed that one panel of figure 4 was from fixed tissue.

10. I accept that the authors did not specifically refer to extrinsic afferent sensitization. My comment was simply a suggestion to assist in interpretation of their observation.

11. Thank you for clarifying the tracing period duration.

## References

[B1] Alagiri M, Chottiner S, Ratner V, Slade D, Hanno PM (1997) Interstitial cystitis: unexplained associations with other chronic disease and pain syndromes. Urology 49:52–57. 9146002 10.1016/s0090-4295(99)80332-x

[B2] Allen DG, Lamb GD, Westerblad H (2008) Skeletal muscle fatigue: cellular mechanisms. Physiol Rev 88:287–332. 10.1152/physrev.00015.2007 18195089

[B3] Angelow S, Ahlstrom R, Yu AS (2008) Biology of claudins. Am J Physiol Renal Physiol 295:F867–F876. 10.1152/ajprenal.90264.2008 18480174 PMC2576152

[B4] Bahns E, Ernsberger U, Jänig W, Nelke A (1986) Functional characteristics of lumbar visceral afferent fibres from the urinary bladder and the urethra in the cat. Pflugers Arch 407:510–518. 3786110 10.1007/BF00657509

[B5] Bahns E, Halsband U, Jänig W (1987) Responses of sacral visceral afferents from the lower urinary tract, colon and anus to mechanical stimulation. Pflugers Arch 410:296–303. 3684516 10.1007/BF00580280

[B6] Barone FC, Deegan JF, Price WJ, Fowler PJ, Fondacaro JD, Ormsbee HS 3rd (1990) Cold-restraint stress increases rat fecal pellet output and colonic transit. The Am J Physiol 258:G329–G337. 2316647 10.1152/ajpgi.1990.258.3.G329

[B7] Bavencoffe A, Li Y, Wu Z, Yang Q, Herrera J, Kennedy EJ, Walters ET, Dessauer CW (2016) Persistent electrical activity in primary nociceptors after spinal cord injury is maintained by scaffolded adenylyl cyclase and protein kinase A and is associated with altered adenylyl cyclase regulation. J Neurosci 36:1660–1668. 10.1523/JNEUROSCI.0895-15.2016 26843647 PMC4737775

[B8] Bedi SS, Yang Q, Crook RJ, Du J, Wu Z, Fishman HM, Grill RJ, Carlton SM, Walters ET (2010) Chronic spontaneous activity generated in the somata of primary nociceptors is associated with pain-related behavior after spinal cord injury. J Neurosci 30:14870–14882. 10.1523/JNEUROSCI.2428-10.2010 21048146 PMC3073589

[B9] Bicer F, Altuntas CZ, Izgi K, Ozer A, Kavran M, Tuohy VK, Daneshgari F (2015) Chronic pelvic allodynia is mediated by CCL2 through mast cells in an experimental autoimmune cystitis model. Am J Physiol Renal Physiol 308:F103–F113. 10.1152/ajprenal.00202.2014 25209862 PMC4338923

[B10] Birder LA (2014) Urinary bladder, cystitis and nerve/urothelial interactions. Auton Neurosci 182:89–94. 10.1016/j.autneu.2013.12.005 24412640 PMC3989437

[B11] Birder LA, de Groat WC (1992) Increased c-fos expression in spinal neurons after irritation of the lower urinary tract in the rat. J Neurosci 12:4878–4889. 1464772 10.1523/JNEUROSCI.12-12-04878.1992 PMC6575764

[B12] Buffington CA (2004) Comorbidity of interstitial cystitis with other unexplained clinical conditions. J Urol 172:1242–1248. 15371816 10.1097/01.ju.0000137953.49304.6c

[B13] Burchiel KJ, Russell LC, Lee RP, Sima AA (1985) Spontaneous activity of primary afferent neurons in diabetic BB/Wistar rats. A possible mechanism of chronic diabetic neuropathic pain. Diabetes 34:1210–1213. 4043559 10.2337/diab.34.11.1210

[B14] Carattino MD, Cueva F, Fonovich-De-Schroeder TM, Zuccollo A (2000) Kallikrein-like amidase activity in renal ischemia and reperfusion. Braz J Med Biol Res 33:595–602. 10.1590/S0100-879X2000000500015 10775892

[B15] Carattino MD, Prakasam HS, Ruiz WG, Clayton DR, McGuire M, Gallo LI, Apodaca G (2013) Bladder filling and voiding affect umbrella cell tight junction organization and function. Am J Physiol Renal Physiol 305:F1158–F1168. 10.1152/ajprenal.00282.2013 23884145 PMC3798726

[B16] Chai TC, Keay S (2004) New theories in interstitial cystitis. Nat Clin Pract Urol 1:85–89. 10.1038/ncpuro0057 16474520

[B17] Chaplan SR, Bach FW, Pogrel JW, Chung JM, Yaksh TL (1994) Quantitative assessment of tactile allodynia in the rat paw. J Neurosci Methods 53:55–63. 7990513 10.1016/0165-0270(94)90144-9

[B18] Chaplan SR, Guo HQ, Lee DH, Luo L, Liu C, Kuei C, Velumian AA, Butler MP, Brown SM, Dubin AE (2003) Neuronal hyperpolarization-activated pacemaker channels drive neuropathic pain. J Neurosci 23:1169–1178. 12598605 10.1523/JNEUROSCI.23-04-01169.2003 PMC6742242

[B19] Corrow KA, Vizzard MA (2009) Phosphorylation of extracellular signal-regulated kinases in bladder afferent pathways with cyclophosphamide-induced cystitis. Neuroscience 163:1353–1362. 10.1016/j.neuroscience.2009.07.044 19638304 PMC2760658

[B20] Cruz CD, Cruz F (2007) The ERK 1 and 2 pathway in the nervous system: from basic aspects to possible clinical applications in pain and visceral dysfunction. Curr Neuropharmacol 5:244–252. 10.2174/157015907782793630 19305741 PMC2644492

[B21] Cruz F, Avelino A, Lima D, Coimbra A (1994) Activation of the c-fos proto-oncogene in the spinal cord following noxious stimulation of the urinary bladder. Somatosens Mot Res 11:319–325. 7778409 10.3109/08990229409028876

[B22] Czéh G, Kríz N, Syková E (1981) Extracellular potassium accumulation in the frog spinal cord induced by stimulation of the skin and ventrolateral columns. J Physiol 320:57–72. 10.1113/jphysiol.1981.sp013934 6976435 PMC1244032

[B23] Dai Y, Iwata K, Fukuoka T, Kondo E, Tokunaga A, Yamanaka H, Tachibana T, Liu Y, Noguchi K (2002) Phosphorylation of extracellular signal-regulated kinase in primary afferent neurons by noxious stimuli and its involvement in peripheral sensitization. J Neurosci 22:7737–7745. 12196597 10.1523/JNEUROSCI.22-17-07737.2002 PMC6757977

[B24] Daly DM, Collins VM, Chapple CR, Grundy D (2011) The afferent system and its role in lower urinary tract dysfunction. Curr Opin Urol 21:268–274. 10.1097/MOU.0b013e3283476ea2 21537194

[B25] Dang K, Bielefeldt K, Gebhart GF (2005) Differential responses of bladder lumbosacral and thoracolumbar dorsal root ganglion neurons to purinergic agonists, protons, and capsaicin. J Neurosci 25:3973–3984. 10.1523/JNEUROSCI.5239-04.2005 15829649 PMC6724937

[B26] Dang K, Bielefeldt K, Gebhart GF (2013) Cyclophosphamide-induced cystitis reduces ASIC channel but enhances TRPV1 receptor function in rat bladder sensory neurons. J Neurophysiol 110:408–417. 10.1152/jn.00945.2012 23636721 PMC3727072

[B27] Dang K, Lamb K, Cohen M, Bielefeldt K, Gebhart GF (2008) Cyclophosphamide-induced bladder inflammation sensitizes and enhances P2X receptor function in rat bladder sensory neurons. J Neurophysiol 99:49–59. 10.1152/jn.00211.2007 17959738 PMC2659400

[B28] de Groat WC, Yoshimura N (2009) Afferent nerve regulation of bladder function in health and disease Handb Exp Pharmacol 91-138. 10.1007/978-3-540-79090-7_4 PMC3383010 19655106

[B29] de Groat WC, Wickens C (2013) Organization of the neural switching circuitry underlying reflex micturition. Acta Physiol (Oxf) 207:66–84. 10.1111/apha.12014 23033877 PMC3718009

[B30] DeBerry JJ, Saloman JL, Dragoo BK, Albers KM, Davis BM (2015) Artemin immunotherapy is effective in preventing and reversing cystitis-induced bladder hyperalgesia via TRPA1 regulation. J Pain 16:628–636. 10.1016/j.jpain.2015.03.014 25892657 PMC4489144

[B31] Dixon WJ (1980) Efficient analysis of experimental observations. Annu Rev Pharmacol Toxicol 20:441–462. 10.1146/annurev.pa.20.040180.002301 7387124

[B32] Djouhri L, Koutsikou S, Fang X, McMullan S, Lawson SN (2006) Spontaneous pain, both neuropathic and inflammatory, is related to frequency of spontaneous firing in intact C-fiber nociceptors. J Neurosci 26:1281–1292. 10.1523/JNEUROSCI.3388-05.2006 16436616 PMC6674571

[B33] Dmitrieva N, McMahon SB (1996) Sensitisation of visceral afferents by nerve growth factor in the adult rat. Pain 66:87–97. 8857635 10.1016/0304-3959(96)02993-4

[B34] Floyd K, Hick VE, Morrison JF (1976) Mechanosensitive afferent units in the hypogastric nerve of the cat. J Physiol 259:457–471. 986462 10.1113/jphysiol.1976.sp011476 PMC1309039

[B35] Foster HE Jr, Lake AG (2014) Use of vanilloids in urologic disorders. Prog Drug Res 68:307–317. 24941675 10.1007/978-3-0348-0828-6_13

[B36] Fowler CJ, Griffiths D, de Groat WC (2008) The neural control of micturition. Nat Rev Neurosci 9:453–466. 10.1038/nrn2401 18490916 PMC2897743

[B37] Gabella G, Davis C (1998) Distribution of afferent axons in the bladder of rats. J Neurocytol 27:141–155. 10640174 10.1023/a:1006903507321

[B38] Gao YJ, Ji RR (2009) c-Fos and pERK, which is a better marker for neuronal activation and central sensitization after noxious stimulation and tissue injury? Open Pain J 2:11–17. 10.2174/1876386300902010011 19898681 PMC2773551

[B39] Gold MS, Traub RJ (2004) Cutaneous and colonic rat DRG neurons differ with respect to both baseline and PGE2-induced changes in passive and active electrophysiological properties. J Neurophysiol 91:2524–2531. 10.1152/jn.00866.2003 14736864

[B40] Gonzalez EJ, Merrill L, Vizzard MA (2014) Bladder sensory physiology: neuroactive compounds and receptors, sensory transducers, and target-derived growth factors as targets to improve function. Am J Physiol Regul Integr Comp Physiol 306:R869–R878. 10.1152/ajpregu.00030.2014 24760999 PMC4159737

[B41] Graham E, Chai TC (2006) Dysfunction of bladder urothelium and bladder urothelial cells in interstitial cystitis. Curr Urol Rep 7:440–446. 17052438 10.1007/s11934-006-0051-8

[B42] Guo C, Yang B, Gu W, Peng B, Xia S, Yang F, Wen D, Geng J, Zhang Y, Zheng J (2013) Intravesical resiniferatoxin for the treatment of storage lower urinary tract symptoms in patients with either interstitial cystitis or detrusor overactivity: a meta-analysis. PLoS One 8:e82591. 10.1371/journal.pone.0082591 24376550 PMC3869704

[B43] Häbler HJ, Jänig W, Koltzenburg M (1993) Myelinated primary afferents of the sacral spinal cord responding to slow filling and distension of the cat urinary bladder. J Physiol 463:449–460. 8246192 10.1113/jphysiol.1993.sp019604 PMC1175353

[B44] Hanno PM, Burks DA, Clemens JQ, Dmochowski RR, Erickson D, Fitzgerald MP, Forrest JB, Gordon B, Gray M, Mayer RD, Newman D, Nyberg L Jr, Payne CK, Wesselmann U, Faraday MM; Interstitial Cystitis Guidelines Panel of the American Urological Association Education and Research, Inc. (2011) AUA guideline for the diagnosis and treatment of interstitial cystitis/bladder pain syndrome. J Urol 185:2162–2170. 10.1016/j.juro.2011.03.064 21497847 PMC9341322

[B45] Hauser PJ, Dozmorov MG, Bane BL, Slobodov G, Culkin DJ, Hurst RE (2008) Abnormal expression of differentiation related proteins and proteoglycan core proteins in the urothelium of patients with interstitial cystitis. J Urol 179:764–769. 10.1016/j.juro.2007.09.022 18082196 PMC2652890

[B46] Hauser PJ, VanGordon SB, Seavey J, Sofinowski TM, Ramadan M, Abdullah S, Buffington CA, Hurst RE (2015) Abnormalities in expression of structural, barrier and differentiation related proteins, and chondroitin sulfate in feline and human interstitial cystitis. J Urol 194:571–577. 10.1016/j.juro.2015.01.090 25636658 PMC4699667

[B47] Hayashi Y, Takimoto K, Chancellor MB, Erickson KA, Erickson VL, Kirimoto T, Nakano K, de Groat WC, Yoshimura N (2009) Bladder hyperactivity and increased excitability of bladder afferent neurons associated with reduced expression of Kv1.4 alpha-subunit in rats with cystitis. Am J Physiol Regul Integr Comp Physiol 296:R1661–R1670. 10.1152/ajpregu.91054.2008 19279288 PMC2689836

[B48] Hodgkin AL, Horowicz P (1959) The influence of potassium and chloride ions on the membrane potential of single muscle fibres. J Physiol 148:127–160. 14402240 10.1113/jphysiol.1959.sp006278 PMC1363113

[B49] Howard FM (2003) Chronic pelvic pain. Obstet Gynecol 101:594–611. 12636968 10.1016/s0029-7844(02)02723-0

[B50] Jänig W, Morrison JF (1986) Functional properties of spinal visceral afferents supplying abdominal and pelvic organs, with special emphasis on visceral nociception. Prog Brain Res 67:87–114. 3823484 10.1016/s0079-6123(08)62758-2

[B51] Ji RR, Woolf CJ (2001) Neuronal plasticity and signal transduction in nociceptive neurons: implications for the initiation and maintenance of pathological pain. Neurobiol Dis 8:1–10. 10.1006/nbdi.2000.0360 11162235

[B52] Ji RR, Befort K, Brenner GJ, Woolf CJ (2002) ERK MAP kinase activation in superficial spinal cord neurons induces prodynorphin and NK-1 upregulation and contributes to persistent inflammatory pain hypersensitivity. J Neurosci 22:478–485. 11784793 10.1523/JNEUROSCI.22-02-00478.2002 PMC6758654

[B53] Kato S, Endoh H, Masuhiro Y, Kitamoto T, Uchiyama S, Sasaki H, Masushige S, Gotoh Y, Nishida E, Kawashima H, Metzger D, Chambon P (1995) Activation of the estrogen receptor through phosphorylation by mitogen-activated protein kinase. Science 270:1491–1494. 7491495 10.1126/science.270.5241.1491

[B54] Khandelwal P, Abraham SN, Apodaca G (2009) Cell biology and physiology of the uroepithelium. Am J Physiol Renal Physiol 297:F1477–F1501. 10.1152/ajprenal.00327.2009 19587142 PMC2801337

[B55] Khandelwal P, Ruiz WG, Balestreire-Hawryluk E, Weisz OA, Goldenring JR, Apodaca G (2008) Rab11a-dependent exocytosis of discoidal/fusiform vesicles in bladder umbrella cells. Proc Natl Acad Sci USA 105:15773–15778. 10.1073/pnas.0805636105 18843107 PMC2572972

[B56] Koltzenburg M, Bennett DL, Shelton DL, McMahon SB (1999) Neutralization of endogenous NGF prevents the sensitization of nociceptors supplying inflamed skin. Eur J Neurosci 11:1698–1704. 10215923 10.1046/j.1460-9568.1999.00590.x

[B57] Lai HH, Gardner V, Ness TJ, Gereau RW (2014) Segmental hyperalgesia to mechanical stimulus in interstitial cystitis/bladder pain syndrome: evidence of central sensitization. J Urol 191:1294–1299. 10.1016/j.juro.2013.11.099 24316091 PMC4070875

[B58] Lameris AL, Huybers S, Kaukinen K, Mäkelä TH, Bindels RJ, Hoenderop JG, Nevalainen PI (2013) Expression profiling of claudins in the human gastrointestinal tract in health and during inflammatory bowel disease. Scand J Gastroenterol 48:58–69. 10.3109/00365521.2012.741616 23205909

[B59] Lee JD, Lee MH (2014) Decreased expression of zonula occludens-1 and occludin in the bladder urothelium of patients with interstitial cystitis/painful bladder syndrome. J Formos Med Assoc 113:17–22. 10.1016/j.jfma.2012.03.010 24445008

[B60] Levite M, Cahalon L, Peretz A, Hershkoviz R, Sobko A, Ariel A, Desai R, Attali B, Lider O (2000) Extracellular K(+) and opening of voltage-gated potassium channels activate T cell integrin function: physical and functional association between Kv1.3 channels and beta1 integrins. J Exp Med 191:1167–1176. 10748234 10.1084/jem.191.7.1167 PMC2193178

[B61] Liu L, Ji F, Liang J, He H, Fu Y, Cao M (2012) Inhibition by dexmedetomidine of the activation of spinal dorsal horn glias and the intracellular ERK signaling pathway induced by nerve injury. Brain Res 1427:1–9. 10.1016/j.brainres.2011.08.019 22050961

[B62] Liu MG, Wang RR, Chen XF, Zhang FK, Cui XY, Chen J (2011) Differential roles of ERK, JNK and p38 MAPK in pain-related spatial and temporal enhancement of synaptic responses in the hippocampal formation of rats: multi-electrode array recordings. Brain Res 1382:57–69. 10.1016/j.brainres.2011.01.076 21284942

[B63] Lyu YS, Park SK, Chung K, Chung JM (2000) Low dose of tetrodotoxin reduces neuropathic pain behaviors in an animal model. Brain Res 871:98–103. 10882788 10.1016/s0006-8993(00)02451-3

[B64] Maggi CA, Lecci A, Santicioli P, Del Bianco E, Giuliani S (1992) Cyclophosphamide cystitis in rats: involvement of capsaicin-sensitive primary afferents. J Auton Nerv Syst 38:201–208. 1613209 10.1016/0165-1838(92)90031-b

[B65] Mankertz J, Schulzke JD (2007) Altered permeability in inflammatory bowel disease: pathophysiology and clinical implications. Curr Opin Gastroenterol 23:379–383. 10.1097/MOG.0b013e32816aa392 17545772

[B66] Mantella RC, Rinaman L, Vollmer RR, Amico JA (2003) Cholecystokinin and D-fenfluramine inhibit food intake in oxytocin-deficient mice. Am J Physiol Regul Integr Comp Physiol 285:R1037–R1045. 10.1152/ajpregu.00383.2002 14557235

[B67] Matkó J (2003) K+ channels and T-cell synapses: the molecular background for efficient immunomodulation is shaping up. Trends Pharmacol Sci 24:385–389. 10.1016/S0165-6147(03)00198-6 12915045

[B68] May V, Vizzard MA (2010) Bladder dysfunction and altered somatic sensitivity in PACAP-/- mice. J Urol 183:772–779. 10.1016/j.juro.2009.09.077 20022034 PMC2917789

[B69] Medford-Davis L, Rafique Z (2014) Derangements of potassium. Emerg Med Clin North Am 32:329–347. 10.1016/j.emc.2013.12.005 24766936

[B70] Montalbetti N, Rued AC, Clayton DR, Ruiz WG, Bastacky SI, Prakasam HS, Eaton AF, Kullmann FA, Apodaca G, Carattino MD (2015) Increased urothelial paracellular transport promotes cystitis. Am J Physiol Renal Physiol 309:F1070–F1081. 10.1152/ajprenal.00200.2015 26423859 PMC4683306

[B71] Moutzouris DA, Falagas ME (2009) Interstitial cystitis: an unsolved enigma. Clin J Am Soc Nephrol 4:1844–1857. 10.2215/CJN.02000309 19808225

[B72] Neuhaus J, Schwalenberg T (2012) Intravesical treatments of bladder pain syndrome/interstitial cystitis. Nat Rev Urol 9:707–720. 10.1038/nrurol.2012.217 23183946

[B73] Nickel JC (2002) Interstitial cystitis: characterization and management of an enigmatic urologic syndrome. Rev Urol 4:112–121. 16985667 PMC1475982

[B74] Obata K, Yamanaka H, Dai Y, Mizushima T, Fukuoka T, Tokunaga A, Noguchi K (2004) Activation of extracellular signal-regulated protein kinase in the dorsal root ganglion following inflammation near the nerve cell body. Neuroscience 126:1011–1021. 10.1016/j.neuroscience.2004.04.036 15207334

[B75] Ogawa T, Ishizuka O, Ueda T, Tyagi P, Chancellor MB, Yoshimura N (2015) Current and emerging drugs for interstitial cystitis/bladder pain syndrome (IC/BPS). Expert Opin Emerg Drugs 20:555–570. 10.1517/14728214.2015.1105216 26535808

[B76] Overgaard K, Nielsen OB, Flatman JA, Clausen T (1999) Relations between excitability and contractility in rat soleus muscle: role of the Na+-K+ pump and Na+/K+ gradients. J Physiol 518:215–225. 10373703 10.1111/j.1469-7793.1999.0215r.x PMC2269417

[B77] Parsons CL (2007) The role of the urinary epithelium in the pathogenesis of interstitial cystitis/prostatitis/urethritis. Urology 69:9–16. 10.1016/j.urology.2006.03.084 17462486

[B78] Parsons CL (2011) The role of a leaky epithelium and potassium in the generation of bladder symptoms in interstitial cystitis/overactive bladder, urethral syndrome, prostatitis and gynaecological chronic pelvic pain. BJU Int 107:370–375. 10.1111/j.1464-410X.2010.09843.x 21176078

[B79] Parsons CL, Lilly JD, Stein P (1991) Epithelial dysfunction in nonbacterial cystitis (interstitial cystitis). J Urol 145:732–735. 2005689 10.1016/s0022-5347(17)38437-9

[B80] Renaud JM, Light P (1992) Effects of K+ on the twitch and tetanic contraction in the sartorius muscle of the frog, Rana pipiens. Implication for fatigue in vivo. Can J Physiol Pharmacol 70:1236–1246. 1493591 10.1139/y92-172

[B81] Rinaman L (2003) Hindbrain noradrenergic lesions attenuate anorexia and alter central cFos expression in rats after gastric viscerosensory stimulation. J Neurosci 23:10084–10092. 14602823 10.1523/JNEUROSCI.23-31-10084.2003 PMC6740871

[B82] Roppolo JR, Tai C, Booth AM, Buffington CA, de Groat WC, Birder LA (2005) Bladder Adelta afferent nerve activity in normal cats and cats with feline interstitial cystitis. J Urol 173:1011–1015. 10.1097/01.ju.0000145591.35569.9e 15711367

[B83] Sadler KE, Stratton JM, Kolber BJ (2014) Urinary bladder distention evoked visceromotor responses as a model for bladder pain in mice. J Vis Exp 86. 10.3791/51413 PMC4181312 24798516

[B84] Sanchez Freire V, Burkhard FC, Kessler TM, Kuhn A, Draeger A, Monastyrskaya K (2010) MicroRNAs may mediate the down-regulation of neurokinin-1 receptor in chronic bladder pain syndrome. Am J Pathol 176:288–303. 10.2353/ajpath.2010.090552 20008142 PMC2797891

[B85] Schulzke JD, Ploeger S, Amasheh M, Fromm A, Zeissig S, Troeger H, Richter J, Bojarski C, Schumann M, Fromm M (2009) Epithelial tight junctions in intestinal inflammation. Ann NY Acad Sci 1165:294–300. 10.1111/j.1749-6632.2009.04062.x 19538319

[B86] Sengupta JN, Gebhart GF (1994) Mechanosensitive properties of pelvic nerve afferent fibers innervating the urinary bladder of the rat. J Neurophysiol 72:2420–2430. 7884468 10.1152/jn.1994.72.5.2420

[B87] Shevock PN, Khan SR, Hackett RL (1993) Urinary chemistry of the normal Sprague-Dawley rat. Urol Res 21:309–312. 8279085 10.1007/BF00296826

[B88] Slobodov G, Feloney M, Gran C, Kyker KD, Hurst RE, Culkin DJ (2004) Abnormal expression of molecular markers for bladder impermeability and differentiation in the urothelium of patients with interstitial cystitis. J Urol 171:1554–1558. 10.1097/01.ju.0000118938.09119.a5 15017219

[B89] Studeny S, Cheppudira BP, Meyers S, Balestreire EM, Apodaca G, Birder LA, Braas KM, Waschek JA, May V, Vizzard MA (2008) Urinary bladder function and somatic sensitivity in vasoactive intestinal polypeptide (VIP)-/- mice. J Mol Neurosci 36:175–187. 10.1007/s12031-008-9100-8 18561033 PMC2693375

[B90] Study RE, Kral MG (1996) Spontaneous action potential activity in isolated dorsal root ganglion neurons from rats with a painful neuropathy. Pain 65:235–242. 8826512 10.1016/0304-3959(95)00216-2

[B91] Sweatt JD (2001) The neuronal MAP kinase cascade: a biochemical signal integration system subserving synaptic plasticity and memory. J Neurochem 76:1–10. 10.1046/j.1471-4159.2001.00054.x 11145972

[B92] Sykova E (1981) K+ changes in the extracellular space of the spinal cord and their physiological role. J Exp Biol 95:93–109. 6278046 10.1242/jeb.95.1.93

[B93] Vizzard MA (2000a) Increased expression of spinal cord Fos protein induced by bladder stimulation after spinal cord injury. Am J Physiol Regul Integr Comp Physiol 279:R295–R305. 10896894 10.1152/ajpregu.2000.279.1.R295

[B94] Vizzard MA (2000b) Alterations in spinal cord Fos protein expression induced by bladder stimulation following cystitis. Am J Physiol Regul Integr Comp Physiol 278:R1027–R1039. 10749792 10.1152/ajpregu.2000.278.4.R1027

[B95] Weber CR, Nalle SC, Tretiakova M, Rubin DT, Turner JR (2008) Claudin-1 and claudin-2 expression is elevated in inflammatory bowel disease and may contribute to early neoplastic transformation. Lab Invest 88:1110–1120. 10.1038/labinvest.2008.78 18711353 PMC2586671

[B96] Wen J, Morrison JF (1995) The effects of high urinary potassium concentration on pelvic nerve mechanoreceptors and 'silent' afferents from the rat bladder. Adv Exp Med Biol 385:237–239. 8571836 10.1007/978-1-4899-1585-6_29

[B97] Xiao WH, Bennett GJ (1995) Synthetic omega-conopeptides applied to the site of nerve injury suppress neuropathic pains in rats. J Pharmacol Exp Ther 274:666–672. 7636726

[B98] Xiao WH, Bennett GJ (2007) Persistent low-frequency spontaneous discharge in A-fiber and C-fiber primary afferent neurons during an inflammatory pain condition. Anesthesiology 107:813–821. 10.1097/01.anes.0000286983.33184.9c 18073557

[B99] Xie W, Strong JA, Meij JT, Zhang JM, Yu L (2005) Neuropathic pain: early spontaneous afferent activity is the trigger. Pain 116:243–256. 10.1016/j.pain.2005.04.017 15964687 PMC1343516

[B100] Yang Q, Wu Z, Hadden JK, Odem MA, Zuo Y, Crook RJ, Frost JA, Walters ET (2014) Persistent pain after spinal cord injury is maintained by primary afferent activity. J Neurosci 34:10765–10769. 10.1523/JNEUROSCI.5316-13.2014 25100607 PMC4122805

[B101] Yoshimura N, de Groat WC (1999) Increased excitability of afferent neurons innervating rat urinary bladder after chronic bladder inflammation. J Neurosci 19:4644–4653. 10341262 10.1523/JNEUROSCI.19-11-04644.1999 PMC6782608

[B102] Yoshimura N, Chancellor MB (2003) Neurophysiology of lower urinary tract function and dysfunction. Rev Urol 5 [Suppl 8]:S3–S10. 16985987 PMC1502389

[B103] Yoshimura N, White G, Weight FF, de Groat WC (1996) Different types of Na+ and A-type K+ currents in dorsal root ganglion neurones innervating the rat urinary bladder. J Physiol 494 [Pt 1]:1–16. 10.1113/jphysiol.1996.sp021471 8814602 PMC1160610

[B104] Yoshimura N, Seki S, Chancellor MB, de Groat WC, Ueda T (2002) Targeting afferent hyperexcitability for therapy of the painful bladder syndrome. Urology 59:61–67. 12007524 10.1016/s0090-4295(01)01639-9

[B105] Yoshimura N, Seki S, Erickson KA, Erickson VL, Hancellor MB, de Groat WC (2003) Histological and electrical properties of rat dorsal root ganglion neurons innervating the lower urinary tract. J Neurosci 23:4355–4361. 12764124 10.1523/JNEUROSCI.23-10-04355.2003 PMC6741085

[B106] Yoshimura N, Oguchi T, Yokoyama H, Funahashi Y, Yoshikawa S, Sugino Y, Kawamorita N, Kashyap MP, Chancellor MB, Tyagi P, Ogawa T (2014) Bladder afferent hyperexcitability in bladder pain syndrome/interstitial cystitis. Int J Urol 21 [Suppl 1]:18–25. 10.1111/iju.12308 24807488 PMC4089034

[B107] Yu AS, Cheng MH, Angelow S, Günzel D, Kanzawa SA, Schneeberger EE, Fromm M, Coalson RD (2009) Molecular basis for cation selectivity in claudin-2-based paracellular pores: identification of an electrostatic interaction site. J Gen Physiol 133:111–127. 10.1085/jgp.200810154 19114638 PMC2606938

[B108] Zeissig S, Burgel N, Günzel D, Richter J, Mankertz J, Wahnschaffe U, Kroesen AJ, Zeitz M, Fromm M, Schulzke JD (2007) Changes in expression and distribution of claudin 2, 5 and 8 lead to discontinuous tight junctions and barrier dysfunction in active Crohn's disease. Gut 56:61–72. 10.1136/gut.2006.094375 16822808 PMC1856677

